# Genome sequence data from 17 accessions of *Ensete ventricosum*, a staple food crop for millions in Ethiopia

**DOI:** 10.1016/j.dib.2018.03.026

**Published:** 2018-03-11

**Authors:** Zerihun Yemataw, Sadik Muzemil, Daniel Ambachew, Leena Tripathi, Kassahun Tesfaye, Alemayheu Chala, Audrey Farbos, Paul O’Neill, Karen Moore, Murray Grant, David J. Studholme

**Affiliations:** a Southern Agricultural Research Institute, Areka Agricultural Research Center, P.O. Box 79, Areka, Ethiopia; b Department of Microbial, Cellular and Molecular Biology, Addis Ababa University, AddisAbaba, Ethiopia; c International Institute of Tropical Agriculture, P.O. Box 30709, Nairobi, Kenya; d Addis Ababa University, Institute of Biotechnology, P.O. Box 1176, Addis Ababa, Ethiopia; e Ethiopian Biotechnology Institute, Ministry of Science and Technology, P.O. Box 32853, Addis Ababa, Ethiopia; f Hawassa University, Awassa College of Agriculture, P.O. Box 05, Hawassa, Ethiopia; g Biosciences, University of Exeter, Exeter EX4 4QD, United Kingdom; h Exeter Sequencing Service, University of Exeter, Exeter EX4 4QD, United Kingdom; i Life Sciences, University of Warwick, Coventry CV4 7AL, United Kingdom

## Abstract

We present raw sequence reads and genome assemblies derived from 17 accessions of the Ethiopian orphan crop plant enset (*Ensete ventricosum* (Welw.) Cheesman) using the Illumina HiSeq and MiSeq platforms. Also presented is a catalogue of single-nucleotide polymorphisms inferred from the sequence data at an average density of approximately one per kilobase of genomic DNA.

**Specifications Table**

**Table:** 

Subject area	*Biology*
More specific subject area	*Genomics of crop plants*
Type of data	*Deoxyribonucleic acid (DNA) sequence*
How data was acquired	*Illumina HiSeq. 2500; Illumina MiSeq*
Data format	*Raw sequence reads; genome sequence assemblies*
Experimental factors	*Genomic DNA was extracted from a selection of 15 enset cultivars and two wild accessions*
Experimental features	*Genome sequencing*
Data source location	*Ethiopia*
Data accessibility	*Sequence data are available from the Sequence Read Archive via BioProjects PRJNA344540*https://www.ncbi.nlm.nih.gov/bioproject/?term=PRJNA344540, *PRJNA342253*https://www.ncbi.nlm.nih.gov/bioproject/?term=PRJNA342253, *PRJNA341828*https://www.ncbi.nlm.nih.gov/bioproject/?term=PRJNA341828, *PRJNA252658*https://www.ncbi.nlm.nih.gov/bioproject/?term=PRJNA252658

**Value of the data**

• Here we present the first genome-wide sequence data available for enset accessions cultivated or growing wild in Ethiopia.
• There is potential to exploit genetic diversity (*e.g.* large numbers of single-nucleotide polymorphisms) to generate markers to assist enset selection for key agronomic traits.
• Given the long lifespan of enset, patterns of genetic variation can be used to classify germplasm and to prioritise and select germplasm for use in breeding.

## 1 Data

The data presented here include enset genomic resequencing data, in the form of sequence reads generated using the Illumina massively parallel deoxyribonucleic acid (DNA) sequencing platform. Also included are draft genome assemblies, a catalogue of single-nucleotide polymorphisms (SNPs) inferred from the sequence data, and images of agarose gels containing results of genotyping assays for several SNPs. Enset (*Ensete ventricosum* (Welw.) Cheesman) is a perennial, herbaceous plant belonging to the same botanical family as bananas and plantains, namely the Musaceae [1]. Although it does not yield edible fruits, it is the most important cultivated staple food crop in the highlands of central, south and southwestern Ethiopia with cultural significance [2] as well as a key role in food security [3], [4]. The main food value is in the large starch-rich corm, which can be boiled and consumed in a similar manner to tubers such as potato or can be used to generate a fermented product known as *kocho* [3], [5], [6], [7], [8], [9].

**Fig. 1:**
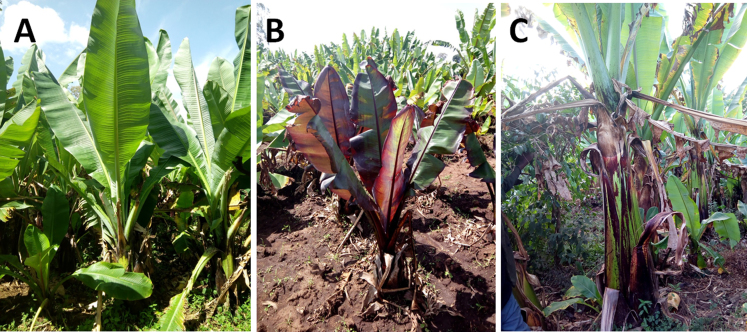
Phenotypic variation among sequenced accessions of *E. ventricosum*. Panels A, B and C shows cultivars Mazia, Lochingie and Nobo respectively.

**Fig. 2:**
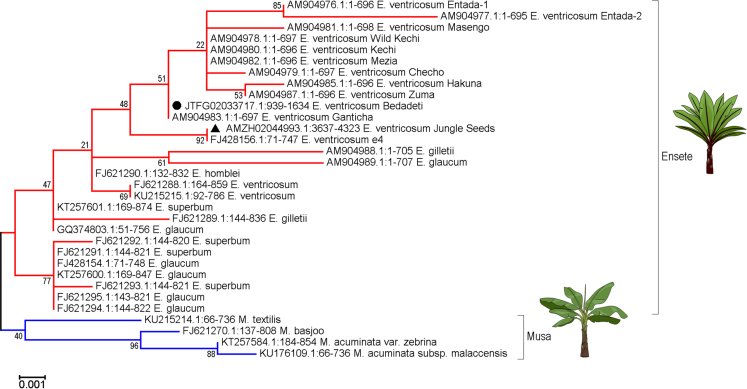
Phylogenetic positions of the enset accessions sequenced here compared to that of the previously sequenced enset genome based on sequences of the *trnF***–***trnT* barcode voucher region of the chloroplast DNA. This locus has previously been used as a barcode and phylogenetic indicator and sequence data for this locus are available from previously published studies (Bekele and Shigeta, [36]; Li et al. [19]; Harrison et al. [18]). There was no sequence variation at this locus among the 17 genomes presented here, as judged by BWA alignments of raw sequence reads against *trnF*-*trnT* sequence. Thus, the branch indicated by the black circle represents the phylogenetic position of all 17 sequenced accessions. A black triangle highlights the position of the “Jungle Seeds” individual whose genome was previously sequenced. The Maximum Likelihood tree presented here is based on a multiple sequence alignment of *trnF*-*trnT* sequences generated using MUSCLE (Edgar, 2004). Evolutionary history was inferred by using the Maximum Likelihood method based on the Tamura-Nei model (Tamura and Nei [37]). The tree with the highest log likelihood (-1249.11) is shown. The percentage of trees in which the associated taxa clustered together is shown next to the branches. Initial tree(s) for the heuristic search were obtained automatically by applying Neighbor-Join and BioNJ algorithms to a matrix of pairwise distances estimated using the Maximum Composite Likelihood (MCL) approach, and then selecting the topology with superior log likelihood value. The tree is drawn to scale, with branch lengths measured in the number of substitutions per site. The analysis involved 32 nucleotide sequences. All positions containing gaps and missing data were eliminated. There were a total of 666 positions in the final dataset. Evolutionary analyses were conducted in MEGA7 (Kumar et al. [38]).

Enset varieties display a great range of genetic and phenotypic variation [7], [10], [11], [12], [13], [14], [15], [16] (Fig. 1) and 15 phenotypic traits have been assayed for a collection of 387 enset accessions [17]. Integration of phenotypic measurements with genetic markers could be of great value in breeding improved varieties with enhanced resistance to abiotic and biotic stresses. Despite its importance for food security of millions in Ethiopia, enset has been relatively neglected in molecular research and few genomic resources are available. We previously published a first draft genome sequence of *E. ventricosum* [18], but the sequenced individual was obtained from the nursery trade (from the UK-based company Jungle Seeds) and its provenance is unknown and therefore its relevance to Ethiopian agriculture is uncertain. Its phylogenetic relationship with Ethiopian varieties is rather distant (Fig. 2), clustering much more closely with *E. ventricosum* e4 (GenBank: FJ428156.1) [19], whose provenance is also unknown. In contrast, the data presented here originate from enset accessions collected in Ethiopia. Most of these enset accessions are sourced from the germplasm collection of the Southern Agricultural Research Institute (SARI), with the exception of Bedadeti, which originated from the collection of the International Institute for Tropical Agriculture (IITA). The data presented here complement previously published genomic resequencing data from *Ensete* species: targetted sequencing of repeats in *Ensete gilletii* [20] and *E. ventricosum* variety Gena [21] and exon sequencing of *Ensete superbum* and *E. ventricosum* [22].

## 2 Experimental design, materials and methods

Genomic DNA was extracted from the young emerging (cigar) leaves using a previously published mini-prep protocol [23]. Between 0.2 and 0.5 g of young and clean leaf was collected per plant and dried in silica gel. From these dried leaves 0.2 g was taken from each sample and ground with sterile pestle and mortar. Genomic DNA was isolated from about 0.2 g of pulverized leaf sample using a modified triple cetyltrimethyl ammonium bromide (CTAB) extraction technique [24]. The yield and quality of DNA were assessed by agarose gel electrophoresis and by a NanoDrop spectrophotometer (NanoDrop Technologies, Wilmington, Delaware) and quantified by Qubit broad range assay (Thermo Fisher Scientific). Illumina sequencing libraries were prepared, after fragmenting 500 ng of DNA to an average size of 500 bp, using Nextflex Rapid DNAseq kit for Illumina sequencing (Bioo Scientific) with adapters containing indexes and 5–8 cycles polymerase chain reaction (PCR) [25]. Library quality was determined using D1000 screen-tapes (Agilent) and libraries were either sequenced individually or combined in equimolar pools.

We sequenced the enset genomic DNA using a combination of Illumina [26], [27] MiSeq and/or Illumina HiSeq. 2500 in either normal or rapid-run modes, as detailed in Table 1. The 17 sequenced accessions included 15 distinct named varieties. We sequenced two different accessions for cultivar Mazia and two different accessions for cultivar Lochingie (a result of complex vernacular naming systems for enset landraces arising from multiple ethno-linguistic communities); one accession was sequenced for each of the other varieties. Raw sequence reads were submitted to the Sequence Read Archive (SRA) [28] under the accession numbers listed in Table 1.

**Table 1:** Illumina sequencing of *E. ventricosum* accessions. Pairs of 100-bp reads were generated using the Illumina HiSeq. 2500 in normal mode except where indicated. A single asterisk (*) indicates use of the Illumina HiSeq. 2500 in rapid-run mode to generate pairs of 300-bp reads and two asterisks (**) indicate use of the Illumina MiSeq to generate pairs of 300-bp reads.

**SARI ID**	**Name**	**Collected from**	**Depth of coverage of genome**	**SRA accession numbers**
362	Arkiya	Dawro	7.36×	SRR4304969, SRR4304970
455	Arkiya	Wolaita	8.04×	SRR4304981*, SRR4304987
112	Astara	Sidama	15.64×	SRR4304989
n/a	Bedadeti	Unknown	45.81×	SRR1515268, SRR1515269**
406	Buffero	West Arsi	18.25×	SRR4304990
435	Derea	Gurage	18.43×	SRR4308285, SRR4308286
451	Erpha 13	Dawro	9.21×	SRR4304991*, SRR4304992
449	Erpha 20	Dawro	9.43×	SRR4304971, SRR4304993*
221	Lochingie	Dawro	8.86×	SRR4304972*, SRR4304973
253	Lochingie	Wolaita	8.66×	SRR4304974*, SRR4304975
208	Mazia	Wolaita	7.00×	SRR4304976*, SRR4304977
429	Mazia	Dawro	8.24×	SRR4304978*, SRR4304979
39	Nechuwe	Gurage	20.69×	SRR4304982
49	Nobo	Sheka	17.16×	SRR4304983
170	Onjamo	Kembata-Tembaro	21.75×	SRR4308284
183	Siyuti	Wolaita	16.54×	SRR4304984
54	Yako	Kaffa	17.96×	SRR4304985

Prior to further analysis, sequence reads were trimmed and filtered using TrimGalore with options “-q 30 --paired”. We performed *de novo* sequence assembly for sequence reads from Bedadeti, Derea and Onjamo (Table 2). For Bedadeti, we used St. Petersburg genome assembler (SPAdes) v. 3.6.1 [29] to assemble contigs and then scaffolded these using Short Sequence Assembly by progressive K-mer search and 3′ read Extension (SSAKE)-based Scaffolding of Pre-Assembled Contigs after Extension (SSPACE) v. 3.0 [30]. For Onjamo, we generated contigs and scaffolds using SPAdes v. 3.9.0 and for Derea generated contigs only using SPAdes v. 3.9.0. SPAdes assemblies were performed using the “--careful” option.

**Table 2:** Assembly statistics for *E. ventricosum* genomes.

GenBank accession number	Enset accession	Total length (bp)	Contig N_50_ (bp)	Scaffold N_50_ (bp)
GCA_000818735.2	Bedadeti	451,284,018	20,943	21,097
GCA_001884805.1	Derea (435)	429,479,738	10,278	n.d.
GCA_001884845.1	Onjamo (170)	444,841,970	15,546	16,208

We identified single-nucleotide polymorphisms by alignment against the reference genome sequence, according to the following procedure. After trimming and filtering with TrimGalore, sequence reads were aligned against the Bedadeti reference genome sequence (GenBank: GCA_000818735.2) using Burrows-Wheeler Aligner (BWA) mem [31], [32] version 0.7.15-r1140 with default options and parameter values.

Candidate SNVs were identified using Sequence Alignment/Map tools (SAMtools)/binary call format tools (BCFtools) package [33], version 1.6, using the following command-lines:

samtools mpileup -u -f genome.fasta alignment.bam > alignment.bcf and.

bcftools call -m -v --Ov alignment.bcf > alignment.vcf

The candidate variants were then filtered using the following command line:

bcftools filter --SnpGap 100 --include '(REF="A" | REF="C" | REF="G" | REF="T") & %QUAL>=35 & MIN(IDV)>=2 & MIN(DP)>=5 & INDEL=0' alignment.vcf > alignment.filtered.vcf

This filtering step eliminates indels with low-confidence single-nucleotide variant calls. It also eliminates candidate SNVs within 10 base pairs of an indel, since alignment artefacts are relatively common in the close vicinity of indels.

**Fig. 3:**
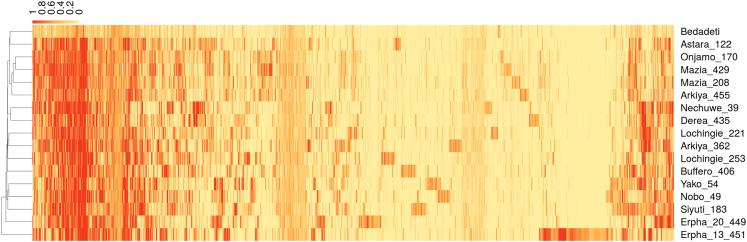
Overview of genetic variation in the sequenced *E. ventricosum* genomes. Each column in the heat-map represents one of 20,000 single-nucleotide variant sites. Each row represents one of the sequenced genomes. Colour indicates the relative frequency of aligned sequence reads with the variant nucleotide at that site in that genome, on a yellow-orange-red palette. Thus, heterozygous sites would be expected to be orange, while homozygous sites would be yellow (same as Bedadeti reference genome sequence) or red (variant from the Bedadeti reference genome sequence). These frequency values were inferred from mpileup-formatted files, generated by aligning genomic sequence reads against the Bedadeti reference genome sequence. The Perl script used to extract these from the mpileup files is included in the Supplementary Material.

Allele frequencies at each SNP site were estimated from frequencies of each base (adenine (A), cytosine (C), guanine (G) or thymine (T)) among the aligned reads. Thus, we would expect an allele frequency of close to zero or one for homozygous sites and approximately 0.5 for heterozygous sites in a diploid genome. The binary alignment/map (BAM)-formatted BWA-mem alignments were converted to pileup format using the *samtools mpileup* command in SAMtools [33] version 1.6 with default options and parameter values. From the resulting pileup files, we used a custom Perl script (included in Supplementary material) to detect SNPs. For SNP detection, we considered only sites where depth of coverage by aligned reads was at least 5× for all 17 datasets. The distribution of a random sample of variants across the 17 accessions is summarized in Fig. 3.

The identification of relatively high-confidence SNPs, distributed throughout the genome at a density of approximately one SNP per kilobase, provides the possibility to develop markers that could be used for genotyping large numbers of plant accessions without the need for large-scale sequencing. One straightforward approach is polymerase chain reaction restriction fragment digest polymorphism (PCR-RFLP) [34]. Another is co-dominant amplified polymorphism (CAPS) [35]. In the PCR-RFLP assay, oligonucleotide primers are designed to amplify a PCR product that flanks a SNP that falls within the recognition site for a restriction enzyme such that one variant is cleavable by the restriction enzyme whilst the other variant is not. Thus, by examining the pattern of bands in agarose electrophoresis of the product after restriction digestion, it is possible to assess the genotype at that SNP location. As a proof of principle, we designed 22 pairs of oligonucleotide primers targeting SNPs identified from the genome sequencing data; these are listed in Table 3. We applied 5 of these assays to several hundred *E. ventricosum* accessions; agarose gels showing the products of digesting the PCR products can be found in the Supplementary material.

**Table 3:** Oligonucleotide primers for PCR-RFLP genotyping assays.

No.	Forward and reverse primer sequences	PCR product size (bp)	Restriction enzyme	Genomic coordinates of PCR target (GenBank accession number: start-end)	Corresponding location in banana genome
1	TAGACTGCCAAGAGACTGCC, GAGTTTGTTCTCCACTTGCTG	395	EcoRV	JTFG02000023: 86778–87172	Chromosome 9
2	CAATGAAATGAGCTCTCGAATGA, CCTCCCTCCCTCTACACAAG	453	ClaI	JTFG02000451: 2383–2835	Chromosome 3
3	AGCTGCCTACTTATGTGCCA, AGGATGGGAGGATTTCACTCA	296	ClaI	JTFG02001079: 44094–44389	No match
4	GAAAGATTCAACCACGCAACA, CAAAGTTGCCCAAATAATAGGGG	100	HindIII	JTFG02001701: 16598–16697	Chromosome 9
5	ACGTAGGAAACAGAAGGCGT, AGAATGAAAACCGGACAGATGA	400	BglII	JTFG02004430: 21696–22095	Chromosome 10
6	GACCAAGGTTGCAACGATGT, AACTCCCTAAAGTGGACCCG	296	HindIII	JTFG02004708: 2865–3160	No match
7	TGCCAATTGTAGCACGCTTT, TCCCAATGATCAGGATGTCATC	321	BglII	JTFG02007725: 4758–5078	Chromosome 4
8	AGCTGATCGGTAGGCTGTTT, TGTTCACTTGCTCAACTTCAATG	329	EcoRV	JTFG02008123: 5568–5896	Chromosome 4
9	CGAAGGAACAAGAGGACGT, CGGCATGAACTAACCGCTTA	380	BglII	JTFG02010045: 2436–2815	No match
10	AGAGTAGAGGTCAGCGCATC, AGGCGAGTGACTAAAGTGCT	385	HindIII	JTFG02015245: 4512–4896	No match
11	GTCATGTAGAATTCAAAAGCCCA, ACCCATGACCAAGACTTTTCT	458	ClaI	JTFG02000797: 35394–35851	Chromosome 10
12	GCAGAATCCCGTGAACCATC, TGTAAGTTTCTTCTCCTCCGCT	377	BglII	JTFG02001387: 44650–45026	Chromosome 10
13	TGCTTTAACCTAGTGAGCTACAA, ACGTCGCCCTTTTACTTTTCT	400	BamHI	JTFG02001793: 29736–30135	Chromosome 7
14	GCCCATGCCATTCTTAAGGA, TCCAATTCCATCCTTCTTCATCT	398	BglII	JTFG02003127: 17456–17853	Matches multiple chromosomes
15	ACTACACAATCCTGGTCCAAAA, CGTAGTTTCCGCCCTTTGAG	113	EcoRV	JTFG02004277: 15220–15332	Chromosome 5
16	CCTGGTTGAGAATGCGGATG, CGACCAATTACACTAAGCCCA	419	BglII	JTFG02006088: 4069–4489	Matches several chromosomes
17	TCCAGCCCAACAATTGATTCTT, CTGAACCTCGGCCAACCT	400	ClaI	JTFG02006206: 13985–14384	Matches several chromosomes
18	TGCCAACCGAACCTCTCAG, TCAGCCATCTACGACATTTACA	400	PstI	JTFG02010369: 10275–10674	No match
19	TGCTTACTGACTATGGAGAGCT, TGCCTGTTTGAGTCCATATAAGT	487	BamHI	JTFG02011833: 6273–6759	Matches several chromosomes
20	CTCGTTAAGGTTCCCCATGC, CCAGCGTGGGAGATCTTTTG	452	EcoRV	JTFG02024842: 425–876	No match
21	CGAGGGCTTCATCGAAAAGG, GCTGCCGACGAGTTGTTC	391	BamHI	JTFG02043259: 629–1019	No match
22	CGATCGTTACGTTGCTTCAG, GGAGCCACAACCAACCAATT	446	PstI	JTFG02009519: 11979–12424	No match

## References

[1] Cheesman E. (1947). Classification of the bananas: the Genus Ensete Horan. Kew Bull..

[2] Tsehaye Y., Kebebew F. (2006). Diversity and cultural use of enset (Enset ventricosum (Welw.) Cheesman) in Bonga in-situ Conservation Site, Ethiopia. Ethnobot. Res. Appl..

[3] Brandt S.A., Spring A., Hiebsch C., McCabe J.T., Tabogie E., Diro M., Wolde-Michael G., Yntiso G., Shigeta M., Tesfaye S. (1997). The “Tree Against Hunger” Enset-based agricultural systems in Ethiopia. Am. Assoc. Adv. Sci..

[4] Negash A., Niehof A. (2004). The significance of enset culture and biodiversity for rural household food and livelihood security in southwestern Ethiopia. Agric. Human. Values.

[5] Yirmaga M.T. (2013). Improving the indigenous processing of kocho, an Ethiopian traditional fermented food. J. Nutr. Food Sci..

[6] Bosha A., Dalbato A.L., Tana T., Mohammed W., Tesfaye B., Karlsson L.M. (2016). Nutritional and chemical properties of fermented food of wild and cultivated genotypes of enset (Ensete ventricosum). Food Res. Int..

[7] Tobiaw (2011). Analysis of genetic diversity among cultivated enset (Ensete ventricosum) populations from Essera and Kefficho, southwestern part of Ethiopia using inter simple sequence repeats (ISSRs) marker. Afr. J. Biotechnol..

[8] Pijls L.T.J., Timmer A.A.M., Wolde-Gebriel Z., West C.E., Pijls C.E., Ainoid T., J., timmer A.M., Wolde-Gwbriel Zewdie (1995). Cultivation, preparation and consumption of ensete (Ensete ventricosum) in Ethiopia. J. Sci. Food Agric..

[9] Bezuxeh T., Feleke A. (1966). The production and utilization of the Genus Ensete in Ethiopia. Econ. Bot..

[10] Tesfaye B., Lüdders P. (2003). Diversity and distribution patterns of enset landraces in Sidama, Southern Ethiopia. Genet. Resour. Crop Evol..

[11] Birmeta G., Nybom H., Bekele E. (2002). RAPD analysis of genetic diversity among clones of the Ethiopian crop plant Ensete ventricosum. Euphytica..

[12] Birmeta G., Nybom H., Bekele E. (2004). Distinction between wild and cultivated enset (Ensete ventricosum) gene pools in Ethiopia using RAPD markers. Hereditas..

[13] Tesfaye B. (2008). On Sidama folk identification, naming, and classification of cultivated enset (Ensete ventricosum) varieties. Genet. Resour. Crop Evol..

[14] Z. Yemataw, H. Mohamed, M. Diro, T. Addis, G. Blomme, Genetic Variability, Inter-Relationships and Path Analysis in Enset ( Ensete ventricosum) Clones, 2012.

[15] Yemataw Z., Mohamed H., Diro M., Addis T., Blomme G. (2014). Ethnic-based diversity and distribution of enset (Ensete ventricosum) clones in southern Ethiopia. J. Ecol. Nat. Environ..

[16] Zippel K. (2005). Diversity Over Time and Space in Enset Landraces (Ensete Ventricosum) in Ethiopia, in: African Biodivers.

[17] Yemataw Z., Chala A., Ambachew D., Studholme D.J., Grant M., Tesfaye K. (2017). Morphological variation and inter-relationships of quantitative traits in enset (Ensete ventricosum (Welw.) Cheesman) germplasm from south and south-western Ethiopia. Plants..

[18] Harrison J., Moore K., Paszkiewicz K., Jones T., Grant M., Ambacheew D., Muzemil S., Studholme D. (2014). A draft genome sequence for Ensete ventricosum, the drought-tolerant “Tree Against Hunger”. Agronomy.

[19] Li L.-F., Häkkinen M., Yuan Y.-M., Hao G., Ge X.-J. (2010). Molecular phylogeny and systematics of the banana family (Musaceae) inferred from multiple nuclear and chloroplast DNA fragments, with a special reference to the genus Musa. Mol. Phylogenet. Evol..

[20] Novák P., Hřibová E., Neumann P., Koblížková A., Doležel J., Macas J. (2014). Genome-wide analysis of repeat diversity across the family Musaceae. PLoS One..

[21] Olango T.M., Tesfaye B., Pagnotta M.A., Pè M.E., Catellani M. (2015). Development of SSR markers and genetic diversity analysis in enset (Ensete ventricosum (Welw.) Cheesman), an orphan food security crop from Southern Ethiopia. BMC Genet..

[22] Sass C., Iles W.J.D., Barrett C.F., Smith S.Y., Specht C.D. (2016). Revisiting the Zingiberales: using multiplexed exon capture to resolve ancient and recent phylogenetic splits in a charismatic plant lineage. Peer J..

[23] Doyle J., Doyle J. (1990). Isolation of plant DNA from fresh tissue. Focus (Madison).

[24] Borsch T., Hilu K.W., Quandt D., Wilde V., Neinhuis C., Barthlott W. (2003). Noncoding plastid trnT-trnF sequences reveal a well resolved phylogeny of basal angiosperms. J. Evol. Biol..

[25] Head S.R., Komori H.K., LaMere S.A., Whisenant T., Van Nieuwerburgh F., Salomon D.R., Ordoukhanian P. (2014). Library construction for next-generation sequencing: overviews and challenges. Biotechniques..

[26] Holt R. a, Jones S.J.M. (2008). The new paradigm of flow cell sequencing. Genome Res..

[27] Mardis E.R. (2013). Next-generation sequencing platforms. Annu. Rev. Anal. Chem..

[28] Leinonen R., Sugawara H., Shumway M. (2011). The sequence read archive. Nucleic Acids Res..

[29] Bankevich A., Nurk S., Antipov D., Gurevich A. a, Dvorkin M., Kulikov A.S., Lesin V.M., Nikolenko S.I., Pham S., Prjibelski A.D., Pyshkin A.V., Sirotkin A.V., Vyahhi N., Tesler G., Alekseyev M.A., Pevzner P.A. (2012). SPAdes: a new genome assembly algorithm and its applications to single-cell sequencing. J. Comput. Biol..

[30] Boetzer M., Henkel C.V., Jansen H.J., Butler D., Pirovano W. (2011). Scaffolding pre-assembled contigs using SSPACE. Bioinformatics..

[31] H. Li, Aligning Sequence Reads, Clone Sequences and Assembly Contigs with BWA-MEM3. 〈http://arxiv.org/abs/1303.3997〉 (Accessed 20 July 2014), 2013.

[32] Li H., Durbin R. (2009). Fast and accurate short read alignment with Burrows-Wheeler transform. Bioinformatics.

[33] Li H., Handsaker B., Wysoker A., Fennell T., Ruan J., Homer N., Marth G., Abecasis G., Durbin R. (2009). 1000 genome project data processing subgroup, the sequence alignment/map format and SAMtools. Bioinformatics.

[34] Pourzand C., Cerutti P. (1993). Genotypic mutation analysis by RFLP/PCR. Mutat. Res..

[35] Konieczny A., Ausubel F.M. (1993). A procedure for mapping Arabidopsis mutations using co-dominant ecotype-specific PCR-based markers. Plant J..

[36] Bekele E., Shigeta M. (2011). Genet. Resour.. Crop Evol..

[37] Tamura K., Nei M. (1993). Estimation of the number of nucleotide substitutions in thecontrol region of mitochondrial DNA in humans and chimpanzees. Mol. Biol.Evol.,.

[38] Kumar S., Stecher G. (2016). K. Tamura, MEGA7: Molecular Evolutionary Genetics AnalysisVersion 7.0 for Bigger Datasets. Mol. Biol. Evol.,.

