# Supplementary material for: Genome sequence data from 17 accessions of Ensete ventricosum, a staple food crop for millions in Ethiopia
Source: Data Brief. 2018 Mar 11;18:285–93. doi: 10.1016/j.dib.2018.03.026 (PMC5996239; doi:10.1016/j.dib.2018.03.026)
Supplement: Supplementary file 1 — Supplementary material [file mmc1.docx]

'Declarations of interest: none'
