# Supplementary material for: Genome sequence data from 17 accessions of Ensete ventricosum, a staple food crop for millions in Ethiopia
Source: Data Brief. 2018 Mar 11;18:285–93. doi: 10.1016/j.dib.2018.03.026 (PMC5996239; doi:10.1016/j.dib.2018.03.026)
Supplement: Supplementary file 3 — Supplementary material [file mmc3.pptx]

## Slide 1
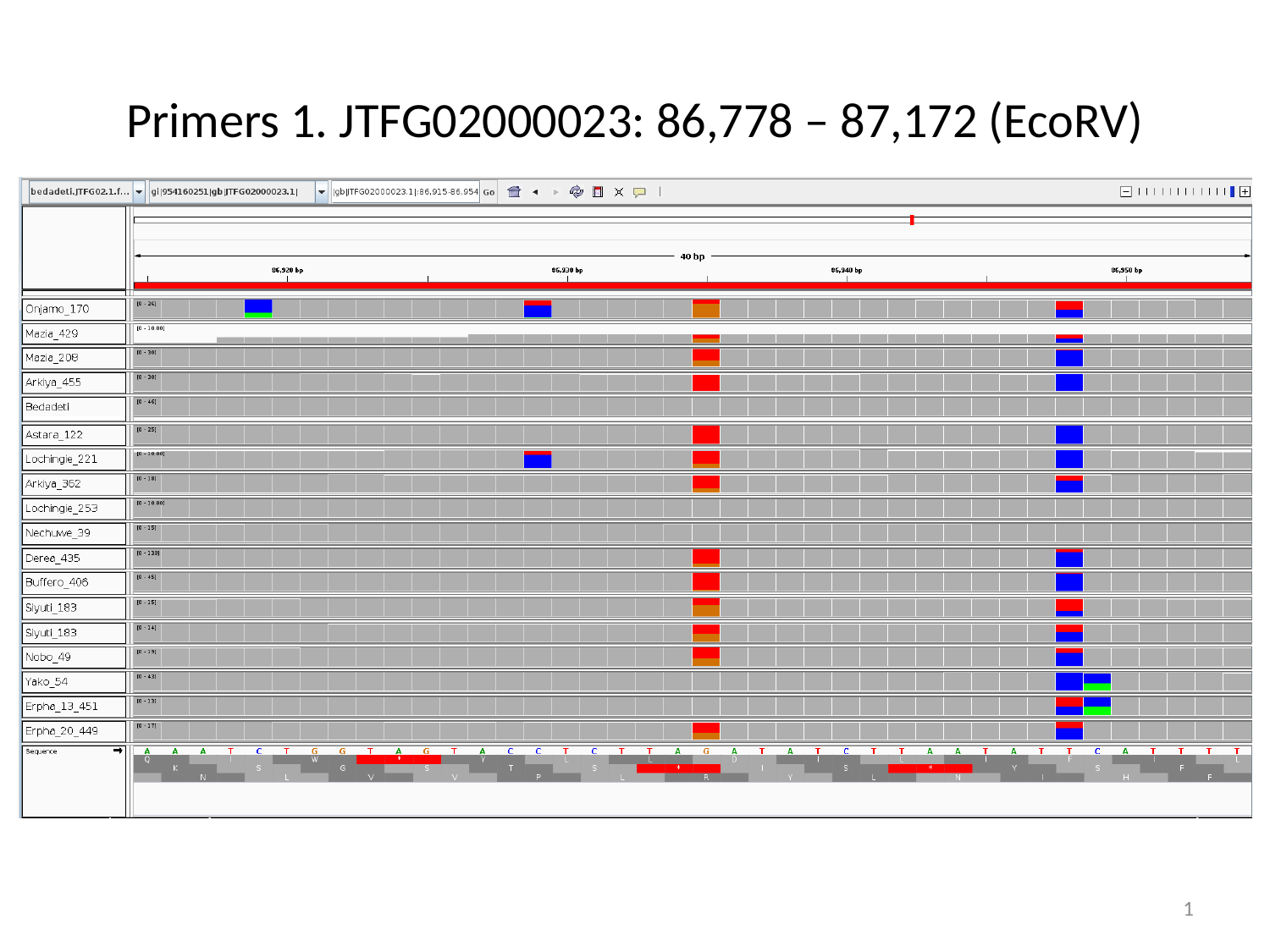

# Primers 1. JTFG02000023: 86,778 – 87,172 (EcoRV)
1

## Slide 2
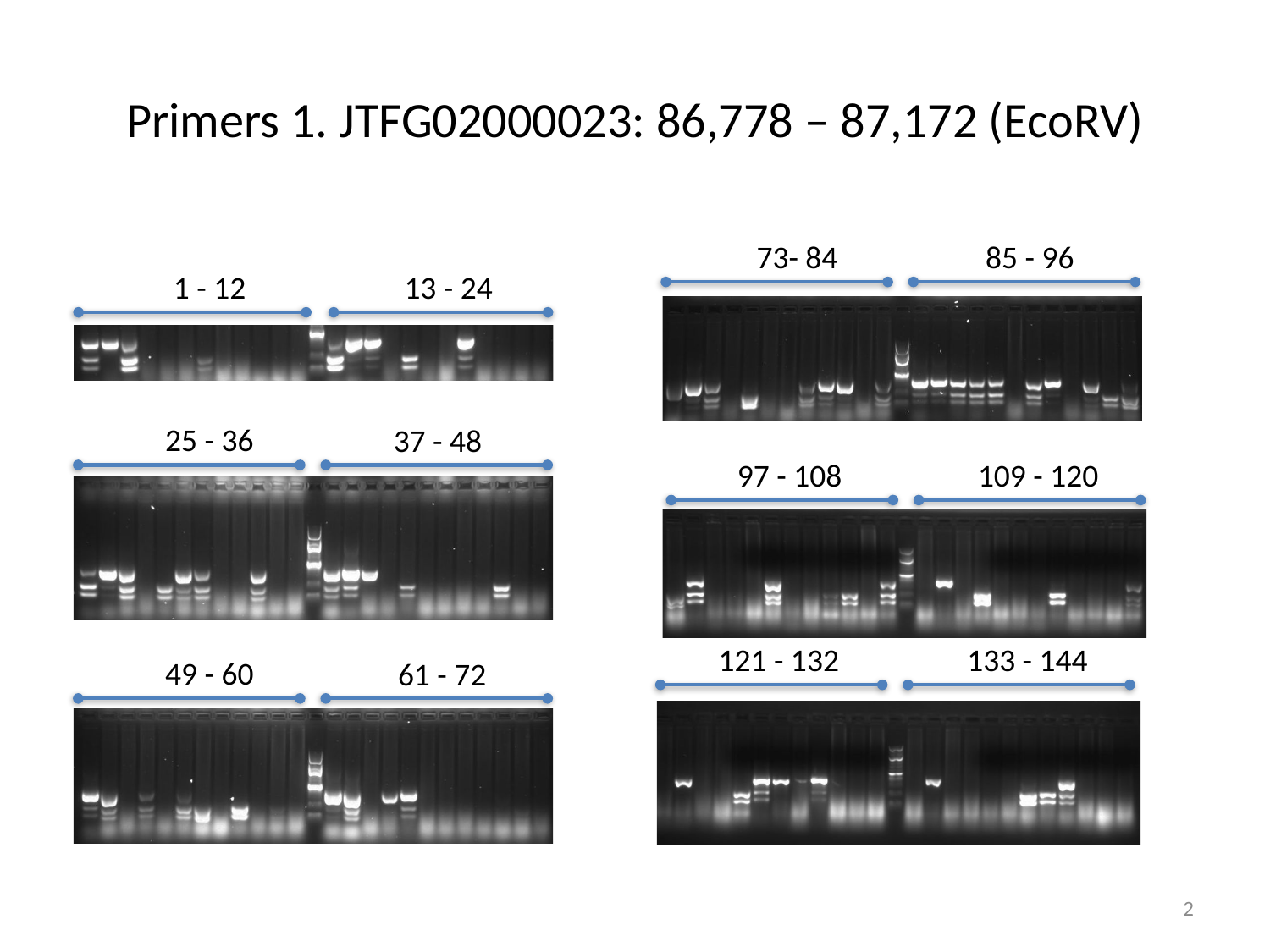

# Primers 1. JTFG02000023: 86,778 – 87,172 (EcoRV)
73- 84
85 - 96
1 - 12
13 - 24
25 - 36
37 - 48
97 - 108
109 - 120
121 - 132
133 - 144
49 - 60
61 - 72
2

## Slide 3
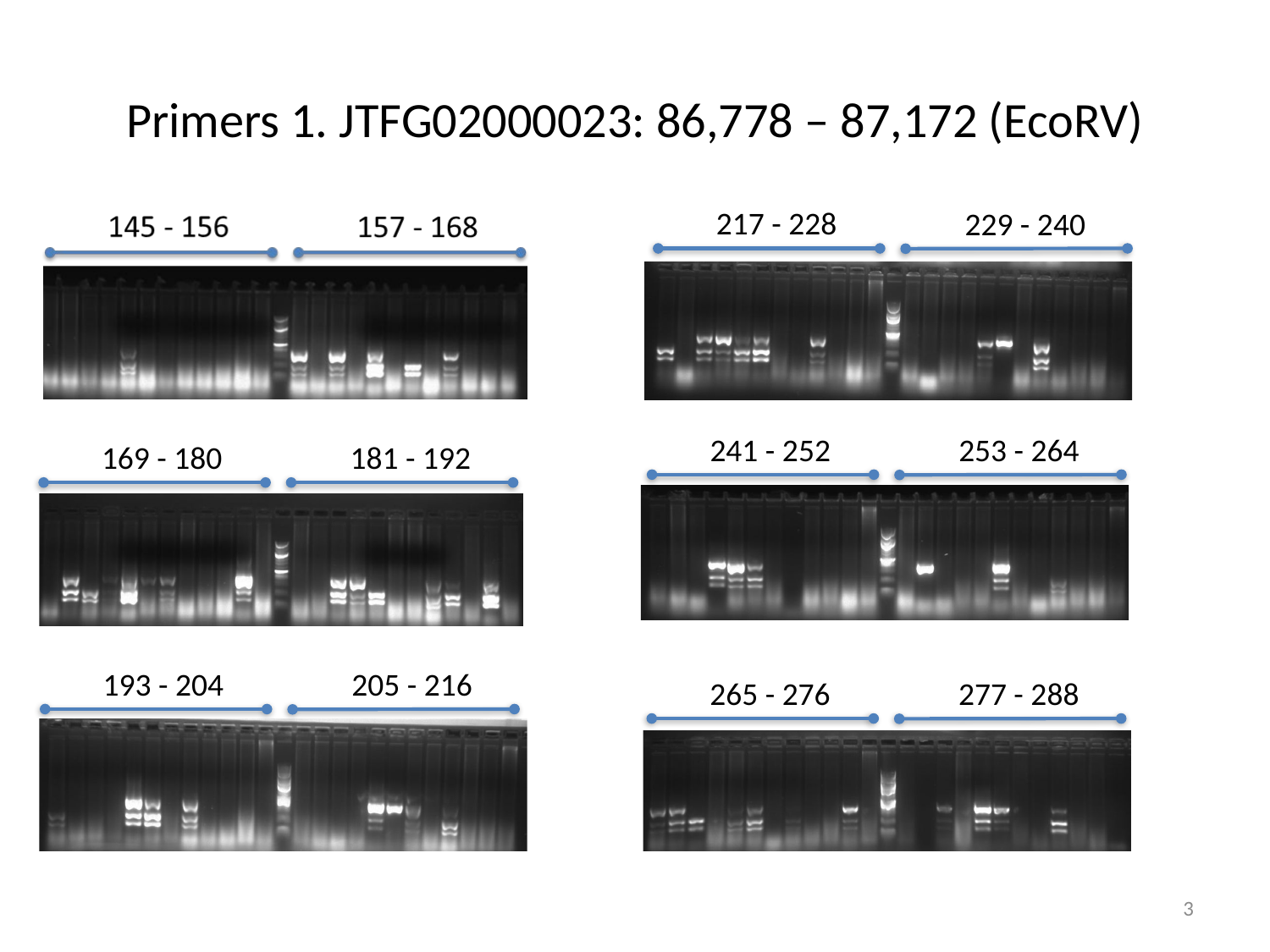

# Primers 1. JTFG02000023: 86,778 – 87,172 (EcoRV)
217 - 228
229 - 240
241 - 252
253 - 264
169 - 180
181 - 192
193 - 204
205 - 216
265 - 276
277 - 288
3

## Slide 4
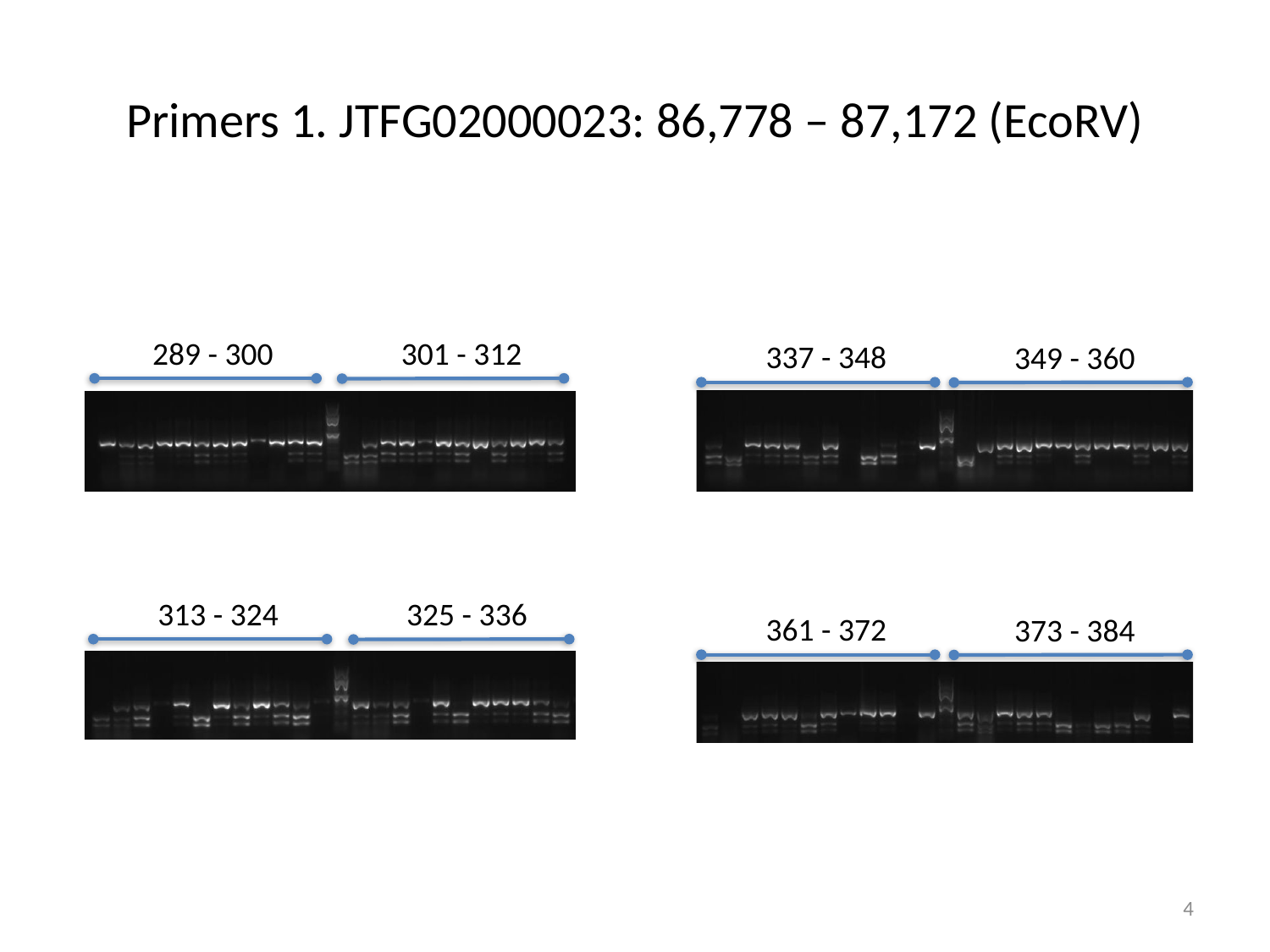

# Primers 1. JTFG02000023: 86,778 – 87,172 (EcoRV)
289 - 300
301 - 312
337 - 348
349 - 360
313 - 324
325 - 336
361 - 372
373 - 384
4

## Slide 5
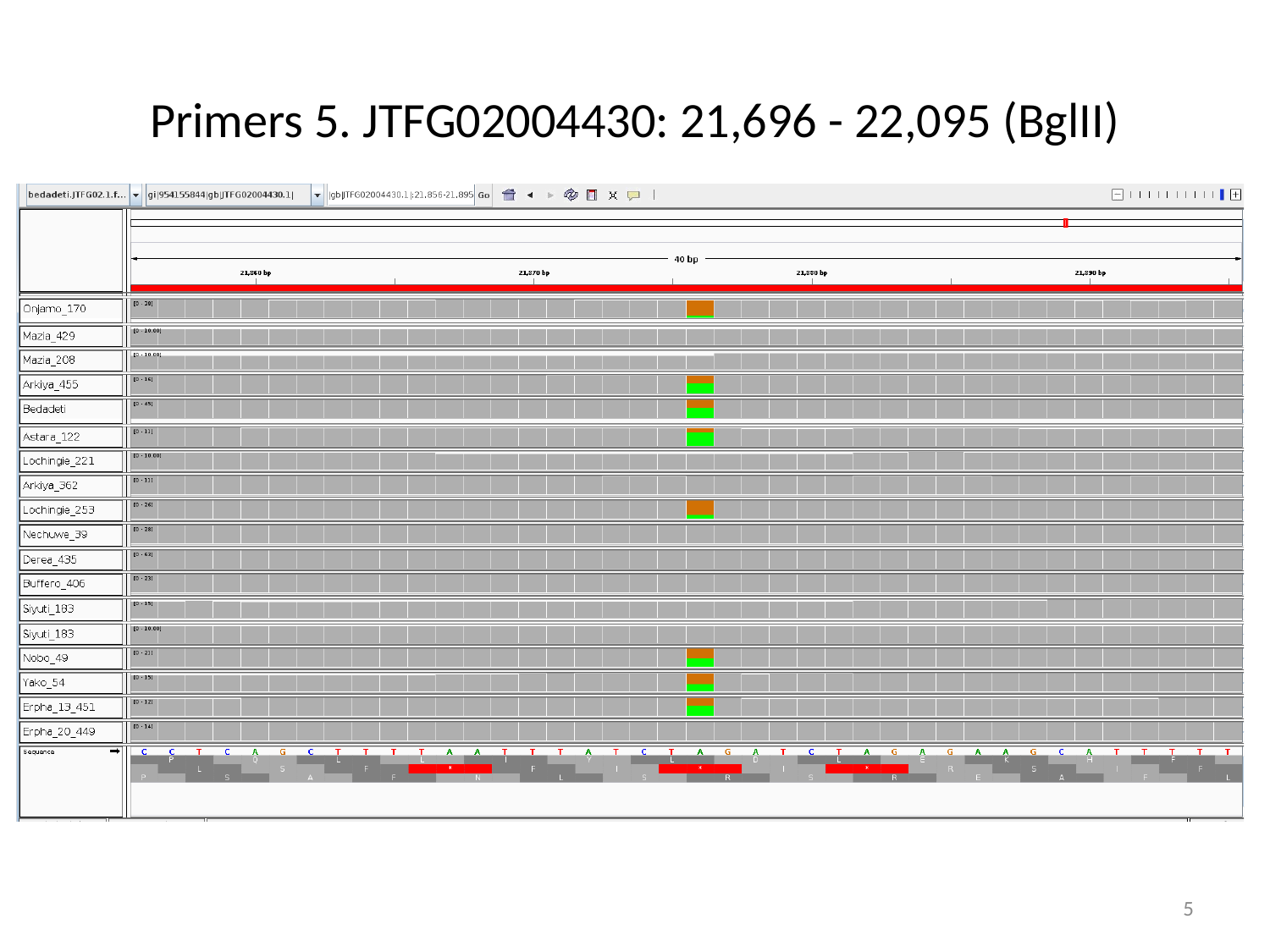

# Primers 5. JTFG02004430: 21,696 - 22,095 (BglII)
5

## Slide 6
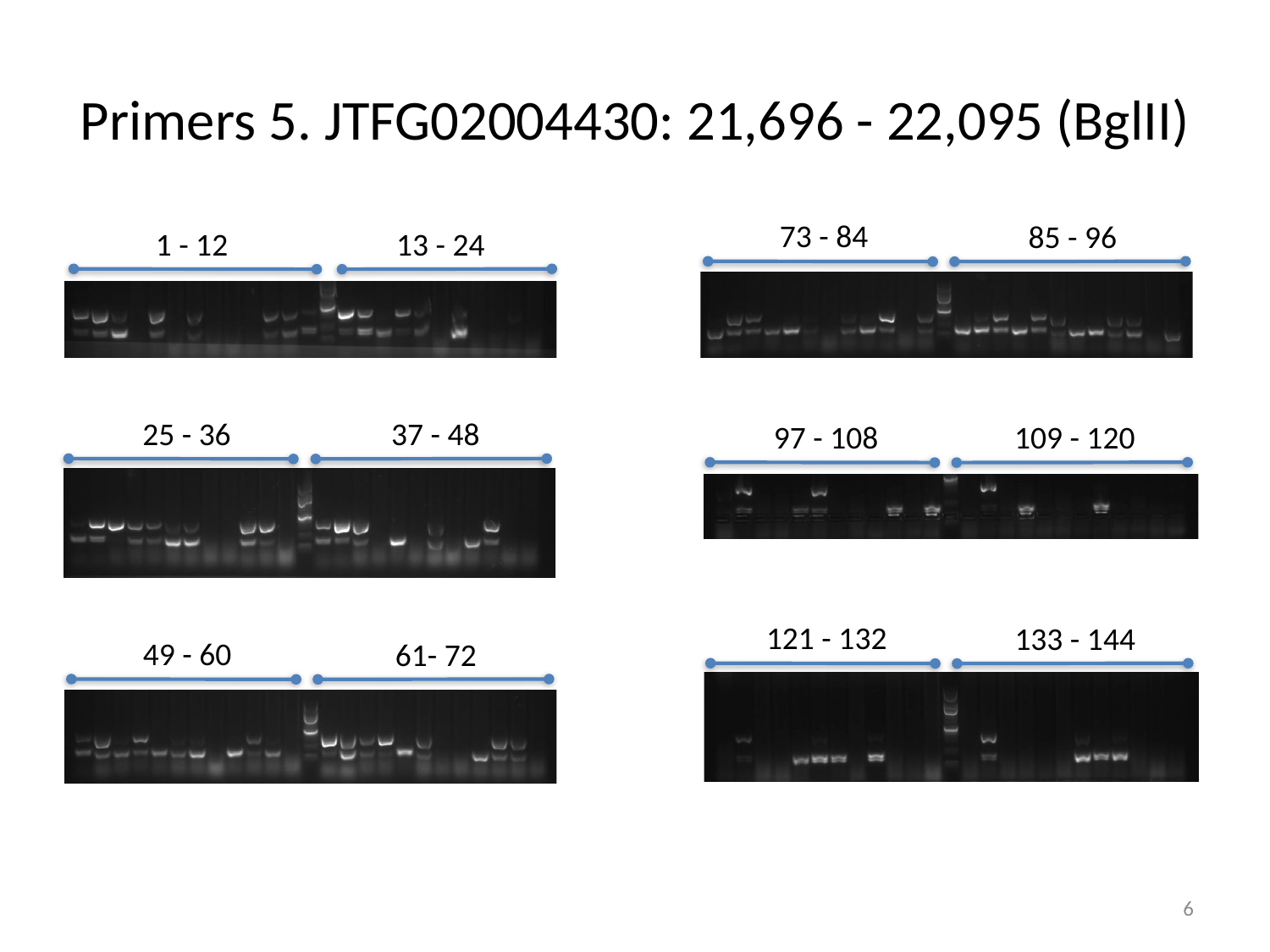

# Primers 5. JTFG02004430: 21,696 - 22,095 (BglII)
73 - 84
85 - 96
1 - 12
13 - 24
25 - 36
37 - 48
97 - 108
109 - 120
121 - 132
133 - 144
49 - 60
61- 72
6

## Slide 7
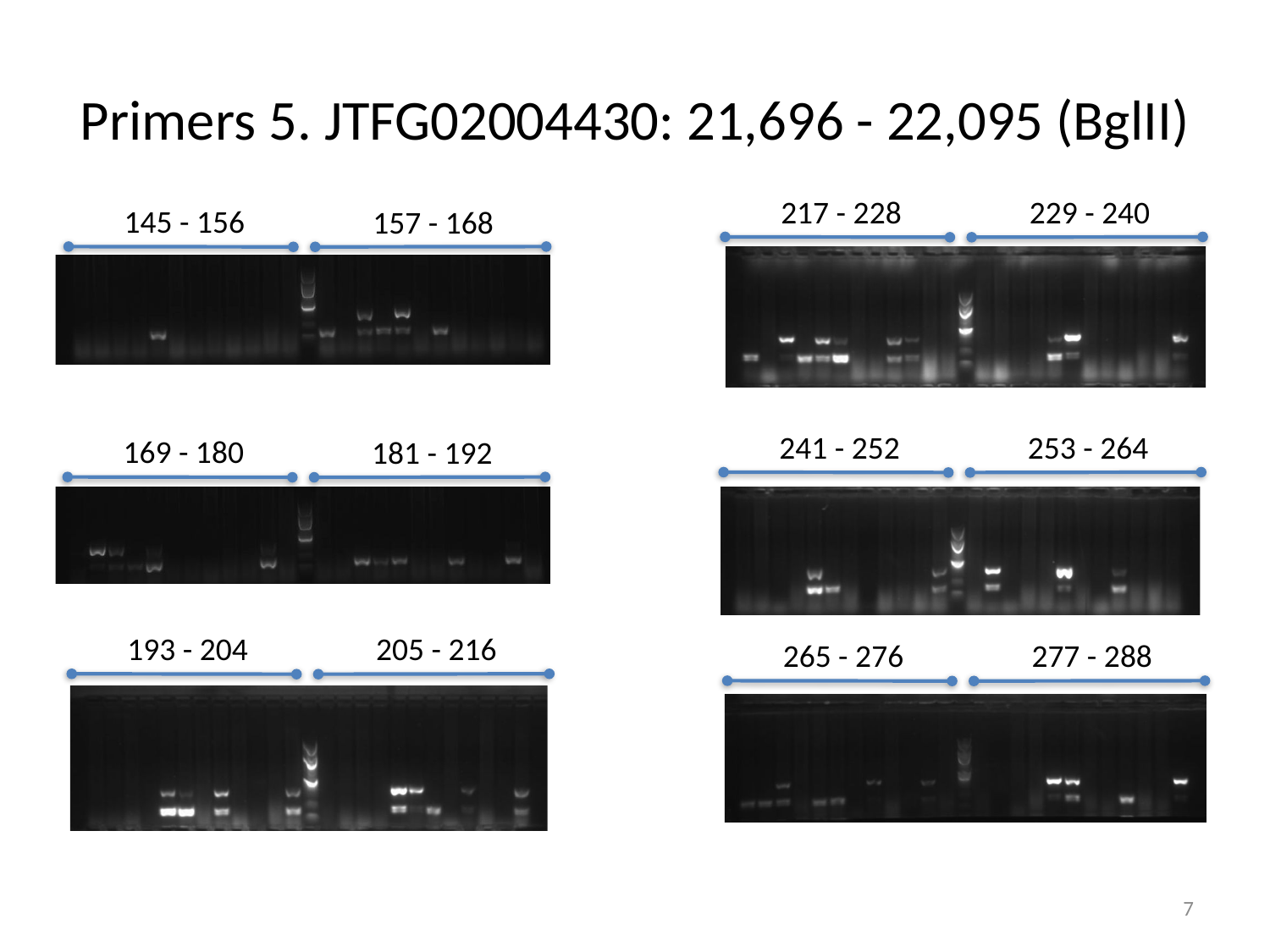

# Primers 5. JTFG02004430: 21,696 - 22,095 (BglII)
217 - 228
229 - 240
145 - 156
157 - 168
241 - 252
253 - 264
169 - 180
181 - 192
193 - 204
205 - 216
265 - 276
277 - 288
7

## Slide 8
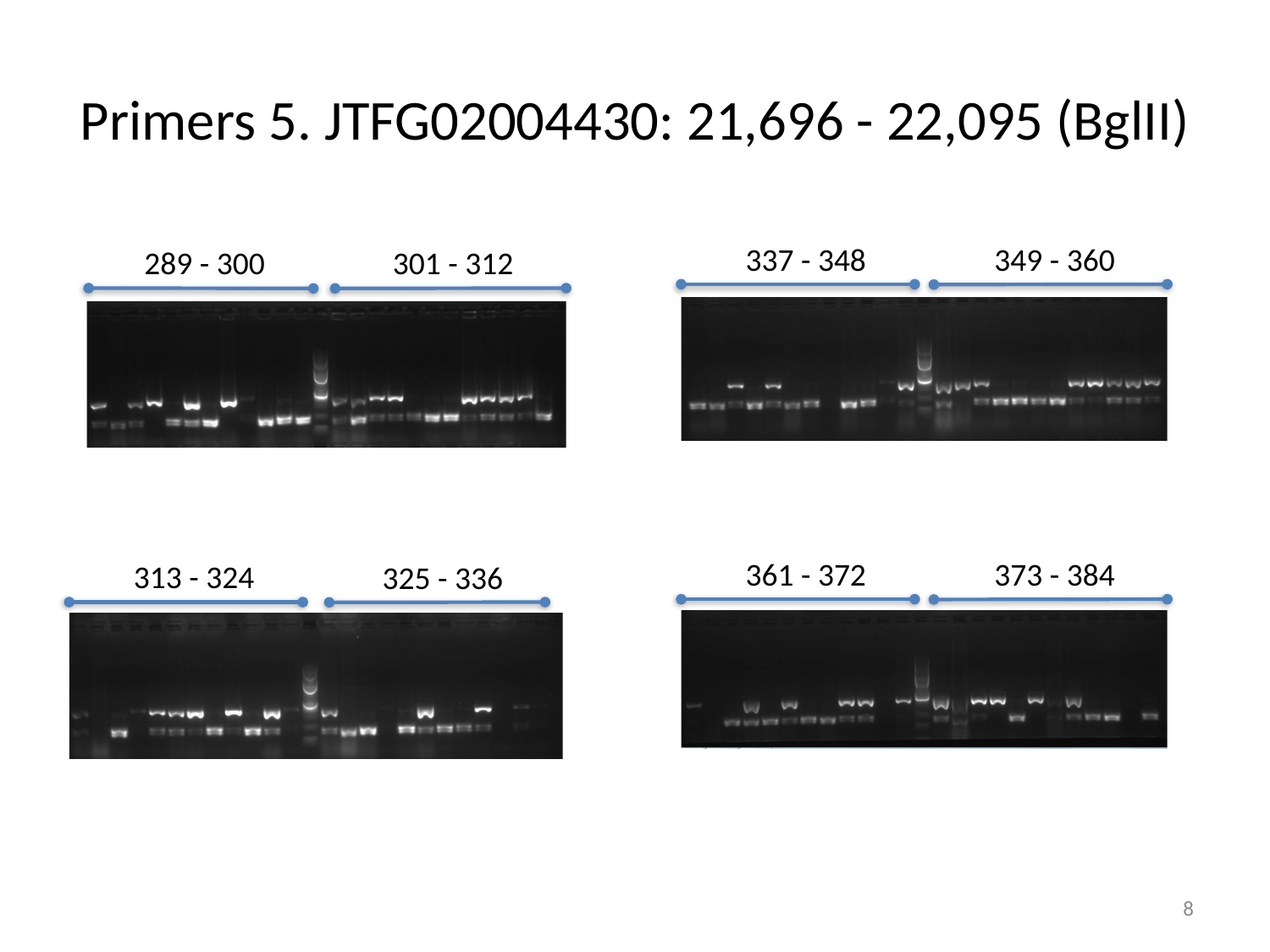

# Primers 5. JTFG02004430: 21,696 - 22,095 (BglII)
337 - 348
349 - 360
289 - 300
301 - 312
361 - 372
373 - 384
313 - 324
325 - 336
8

## Slide 9
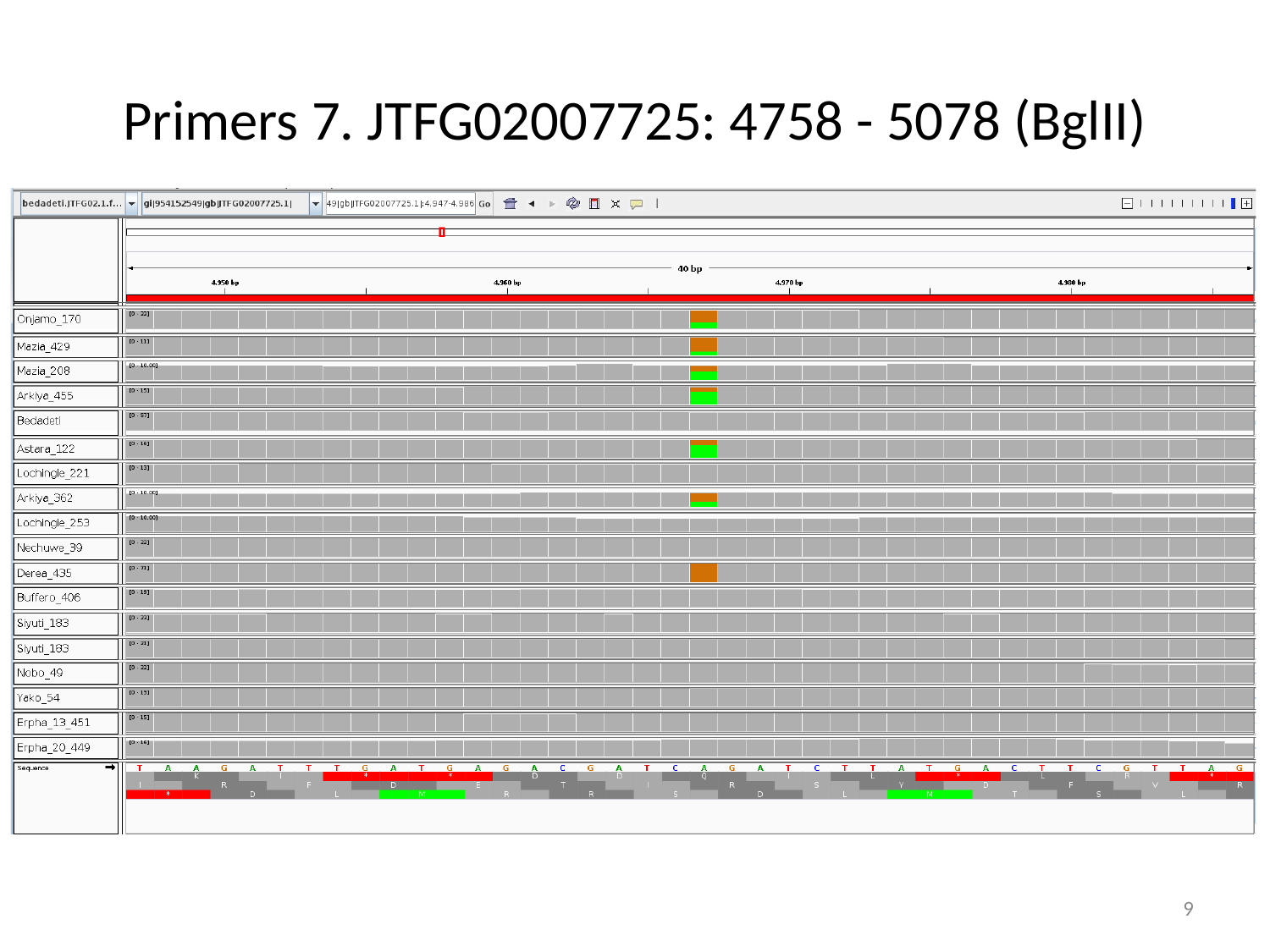

# Primers 7. JTFG02007725: 4758 - 5078 (BglII)
9

## Slide 10
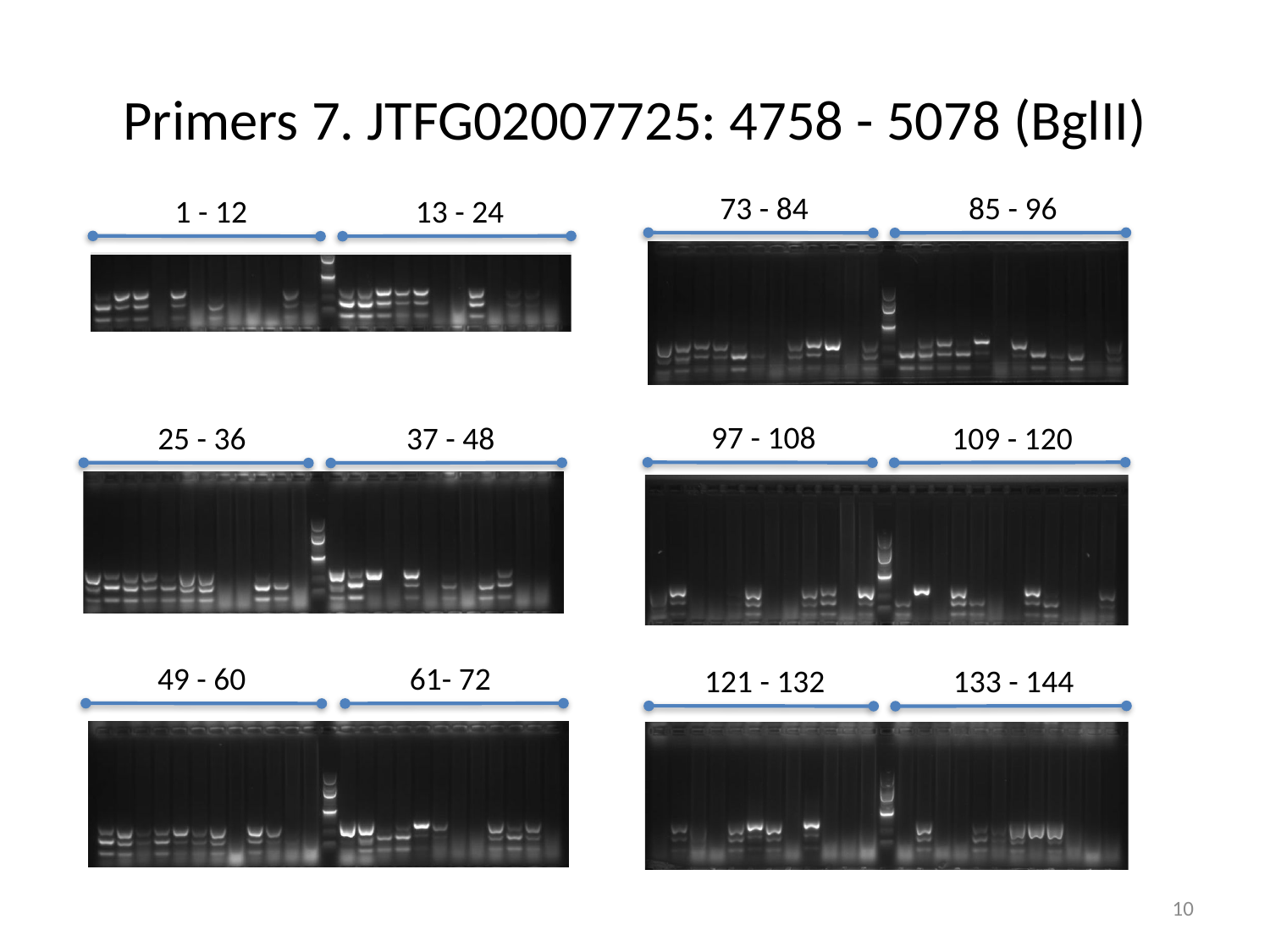

# Primers 7. JTFG02007725: 4758 - 5078 (BglII)
73 - 84
85 - 96
1 - 12
13 - 24
97 - 108
25 - 36
109 - 120
37 - 48
49 - 60
61- 72
121 - 132
133 - 144
10

## Slide 11
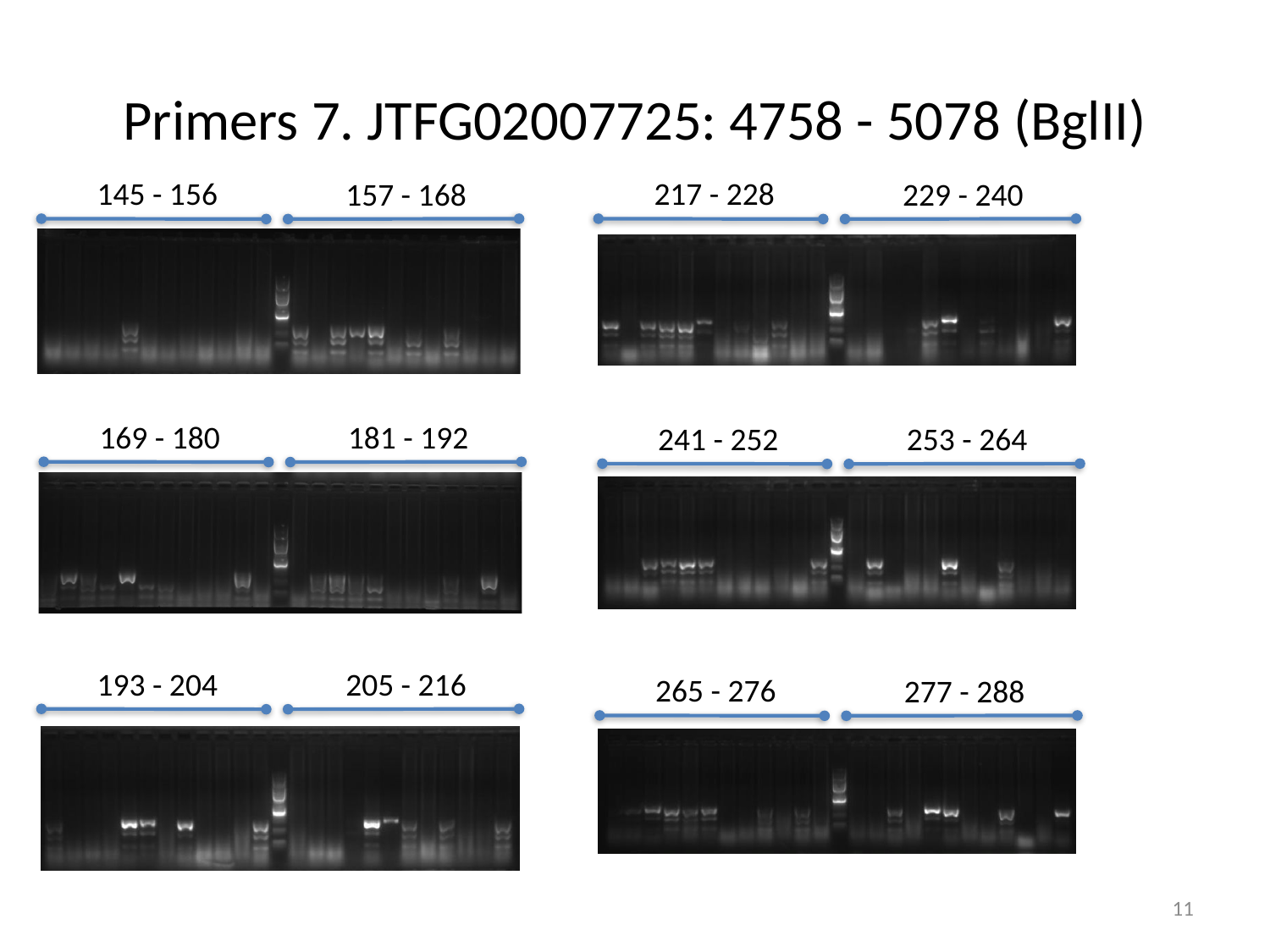

# Primers 7. JTFG02007725: 4758 - 5078 (BglII)
145 - 156
217 - 228
157 - 168
229 - 240
169 - 180
181 - 192
241 - 252
253 - 264
193 - 204
205 - 216
265 - 276
277 - 288
11

## Slide 12
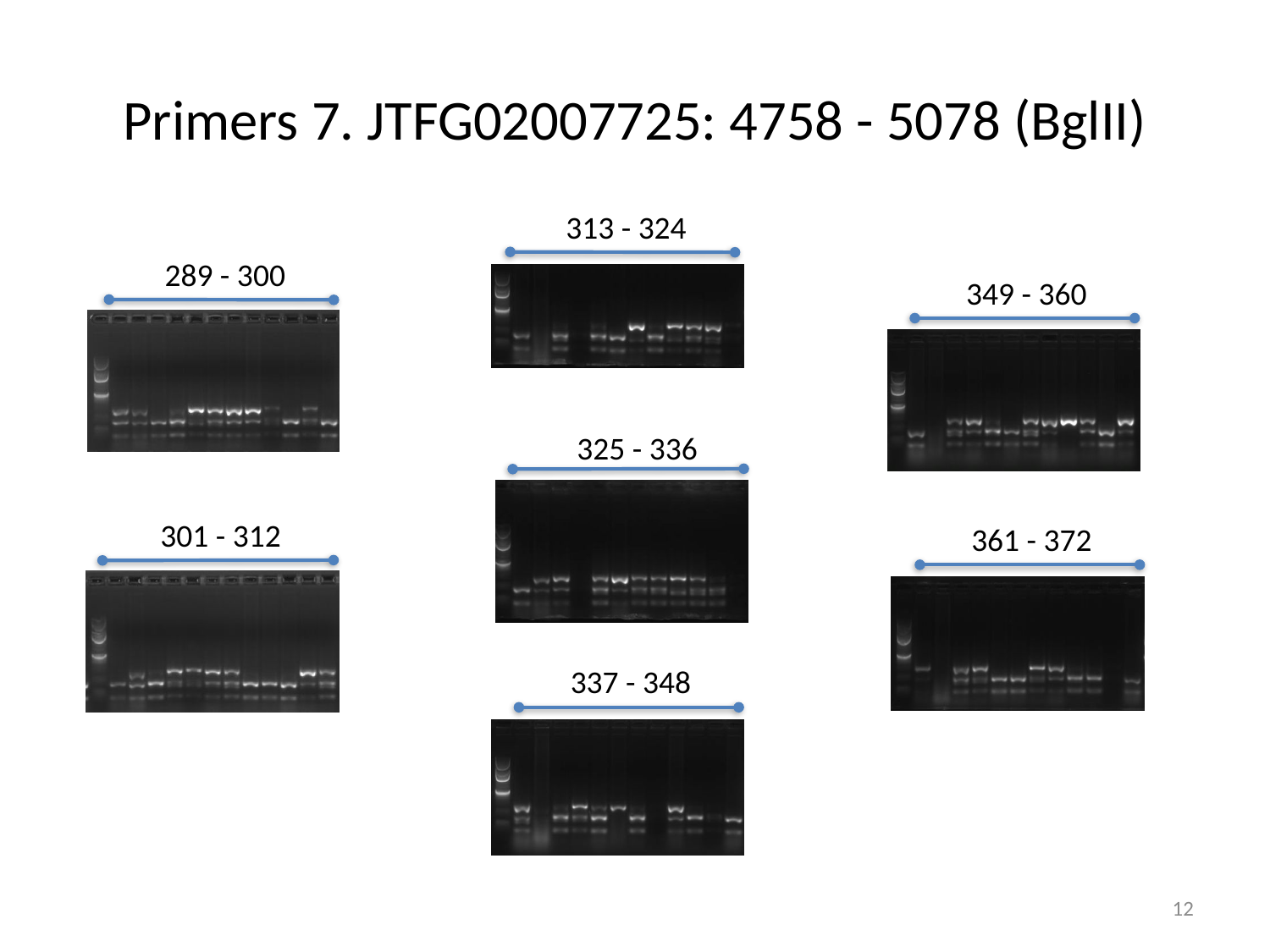

# Primers 7. JTFG02007725: 4758 - 5078 (BglII)
313 - 324
289 - 300
349 - 360
325 - 336
301 - 312
361 - 372
337 - 348
12

## Slide 13
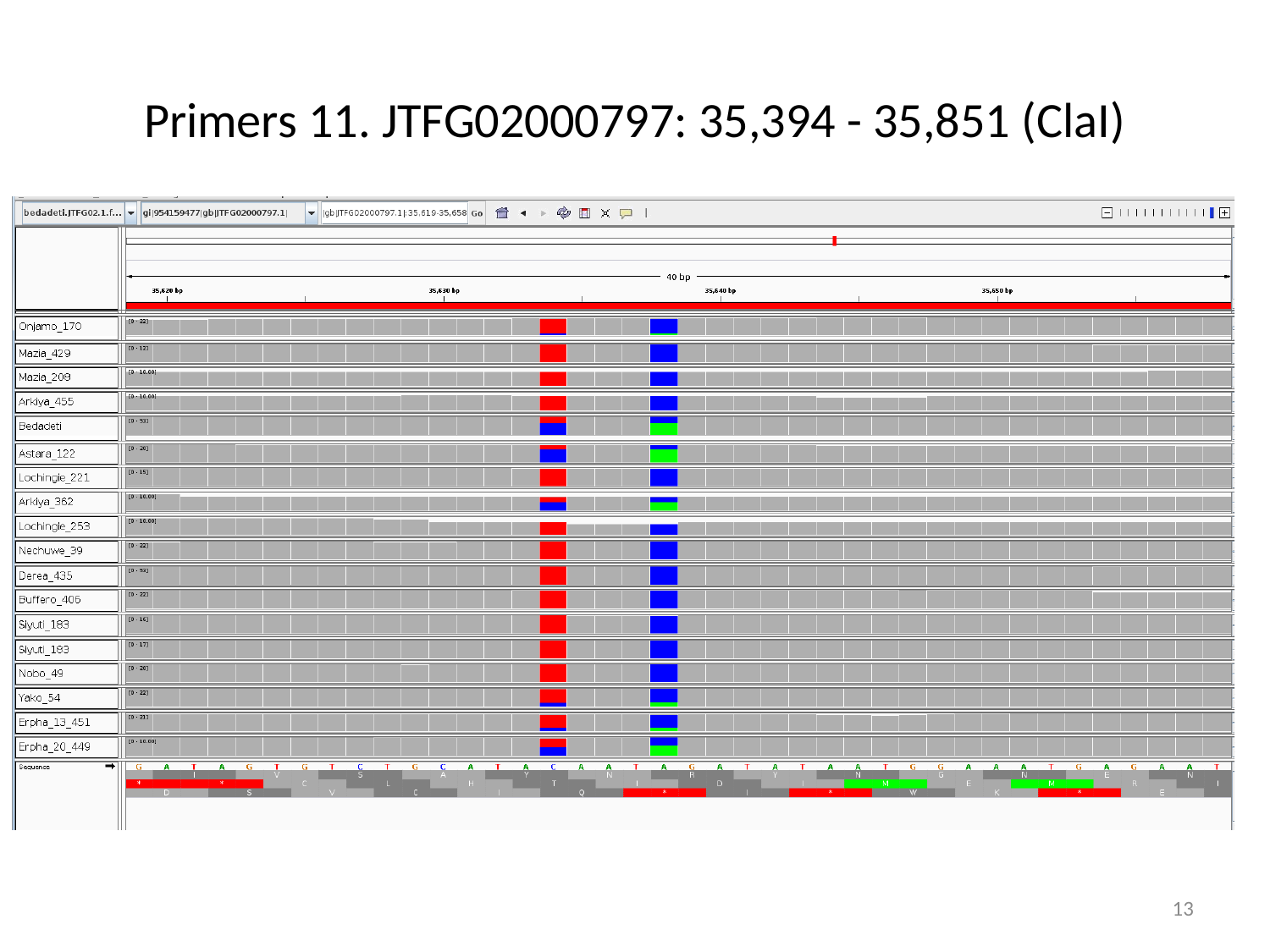

# Primers 11. JTFG02000797: 35,394 - 35,851 (ClaI)
13

## Slide 14
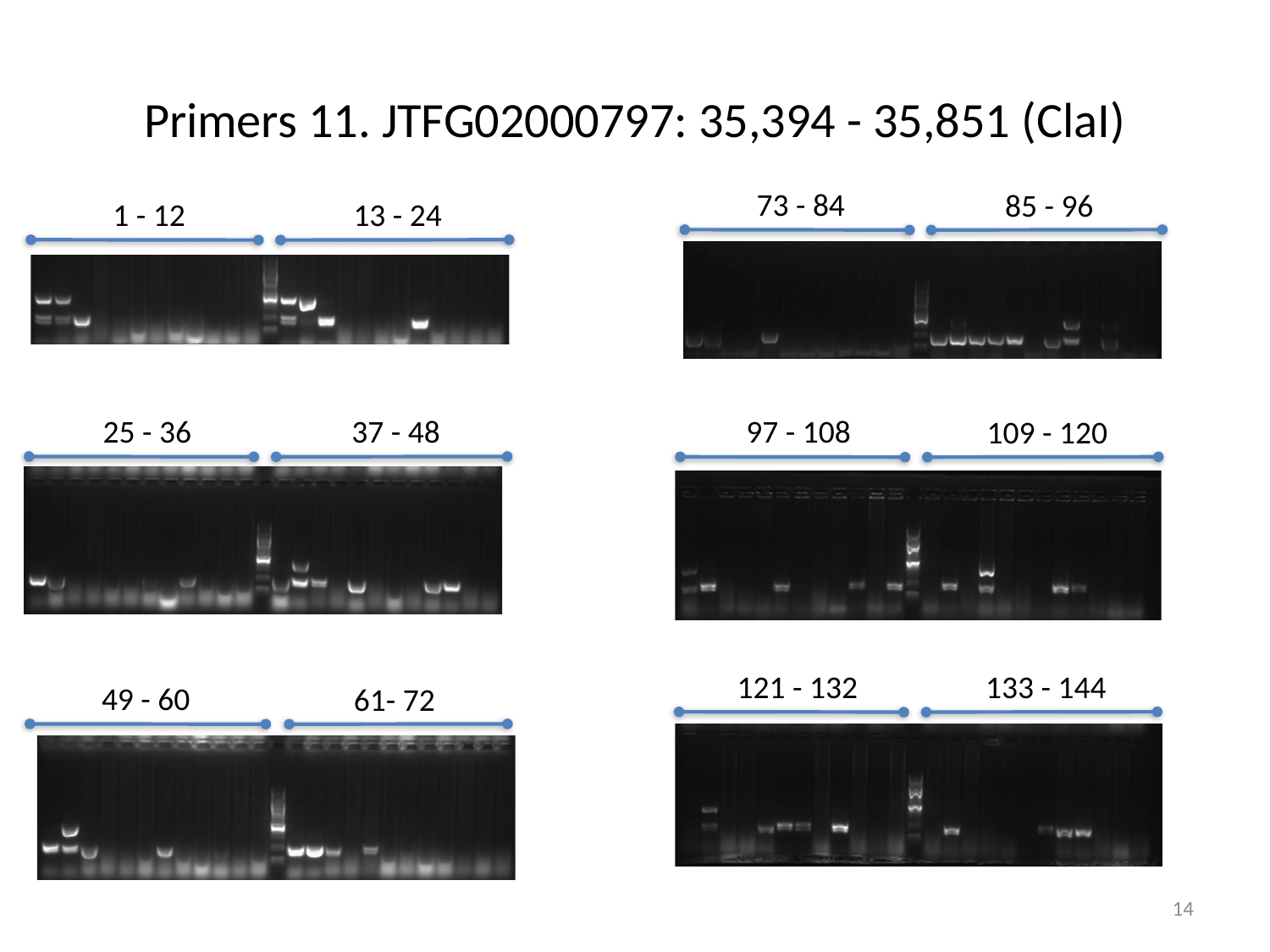

# Primers 11. JTFG02000797: 35,394 - 35,851 (ClaI)
73 - 84
85 - 96
1 - 12
13 - 24
25 - 36
37 - 48
97 - 108
109 - 120
121 - 132
133 - 144
49 - 60
61- 72
14

## Slide 15
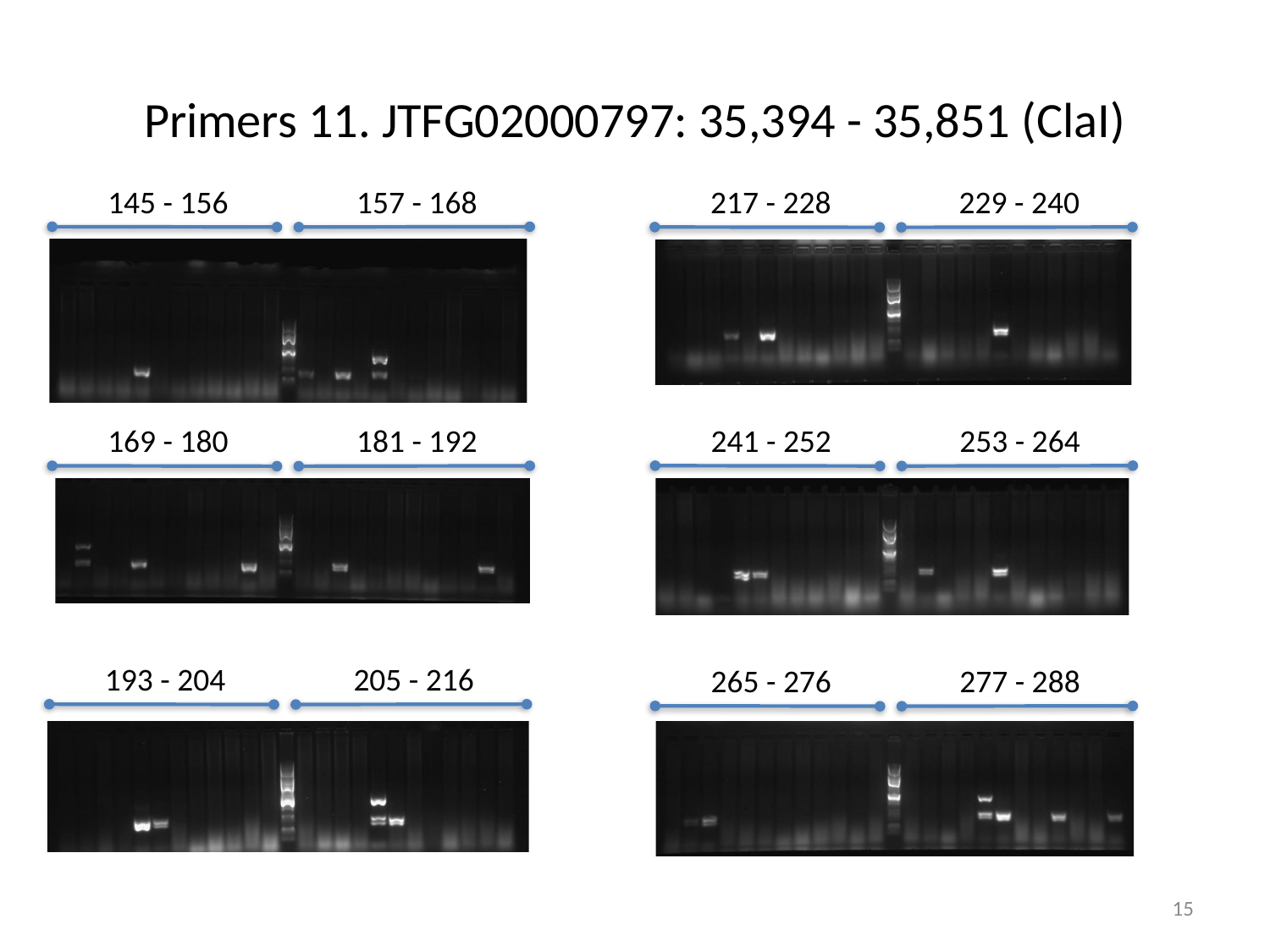

# Primers 11. JTFG02000797: 35,394 - 35,851 (ClaI)
145 - 156
157 - 168
217 - 228
229 - 240
241 - 252
169 - 180
253 - 264
181 - 192
193 - 204
205 - 216
265 - 276
277 - 288
15

## Slide 16
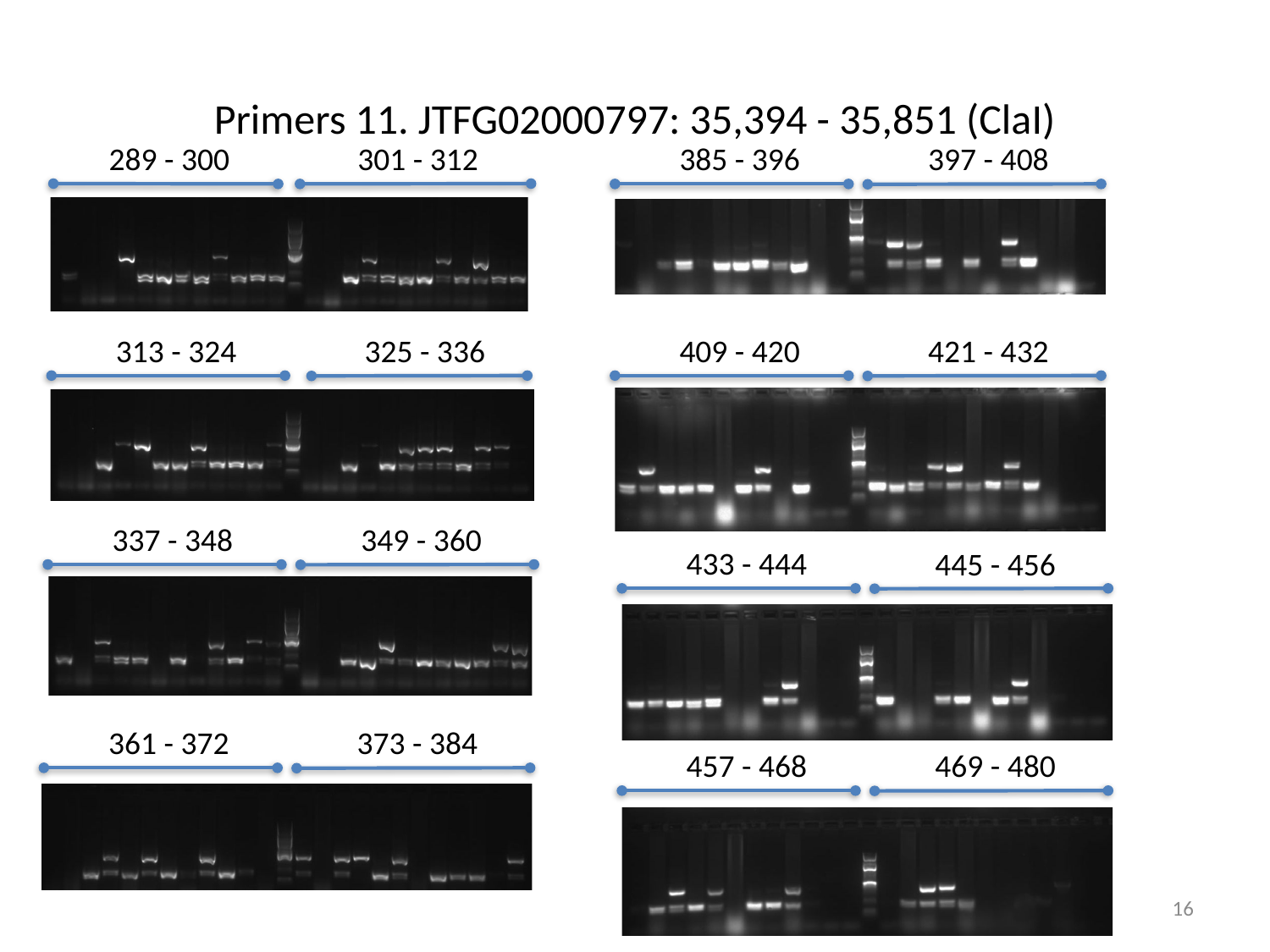

# Primers 11. JTFG02000797: 35,394 - 35,851 (ClaI)
289 - 300
301 - 312
385 - 396
397 - 408
313 - 324
409 - 420
325 - 336
421 - 432
337 - 348
349 - 360
433 - 444
445 - 456
361 - 372
373 - 384
457 - 468
469 - 480
16

## Slide 17
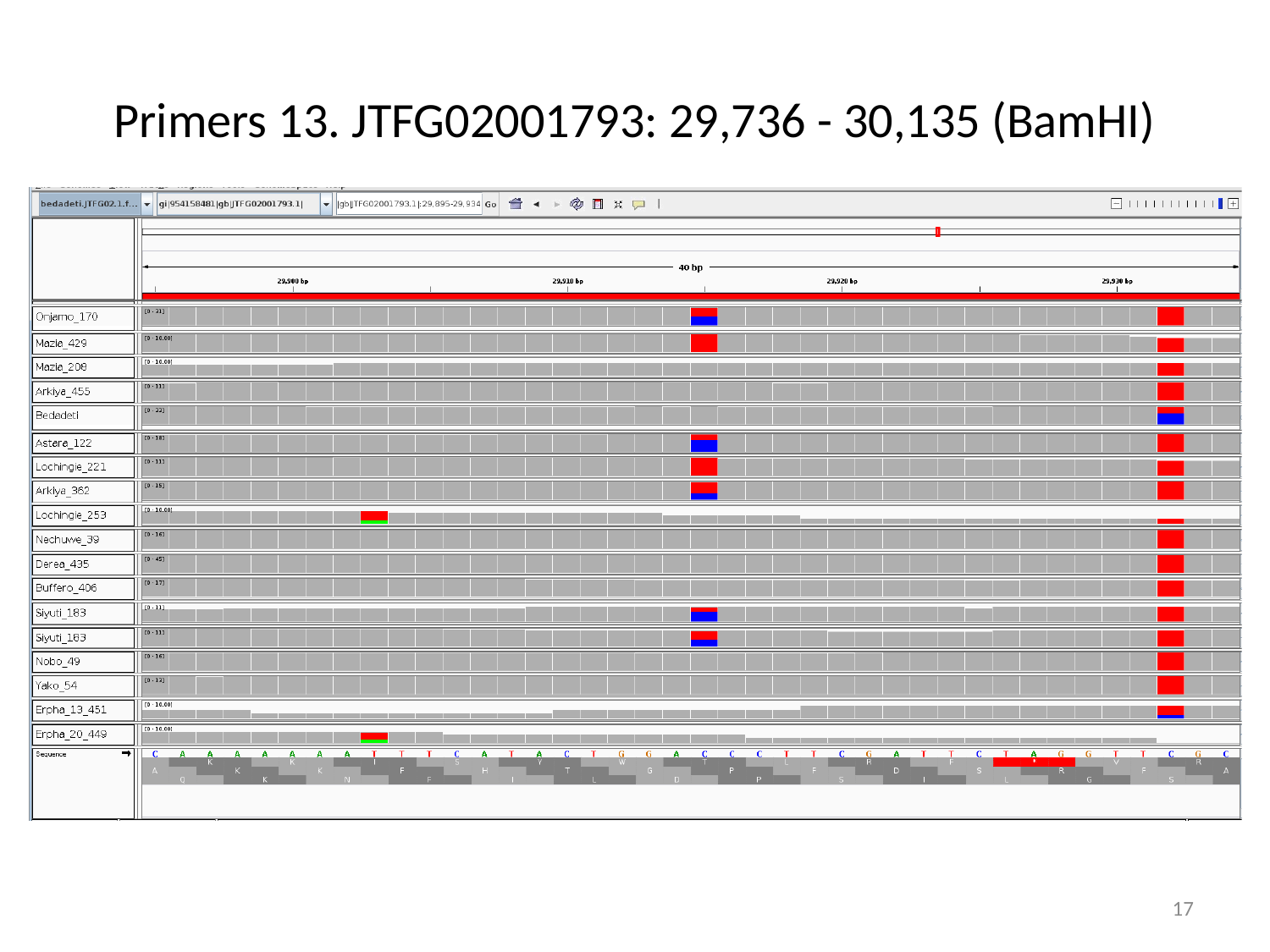

# Primers 13. JTFG02001793: 29,736 - 30,135 (BamHI)
17

## Slide 18
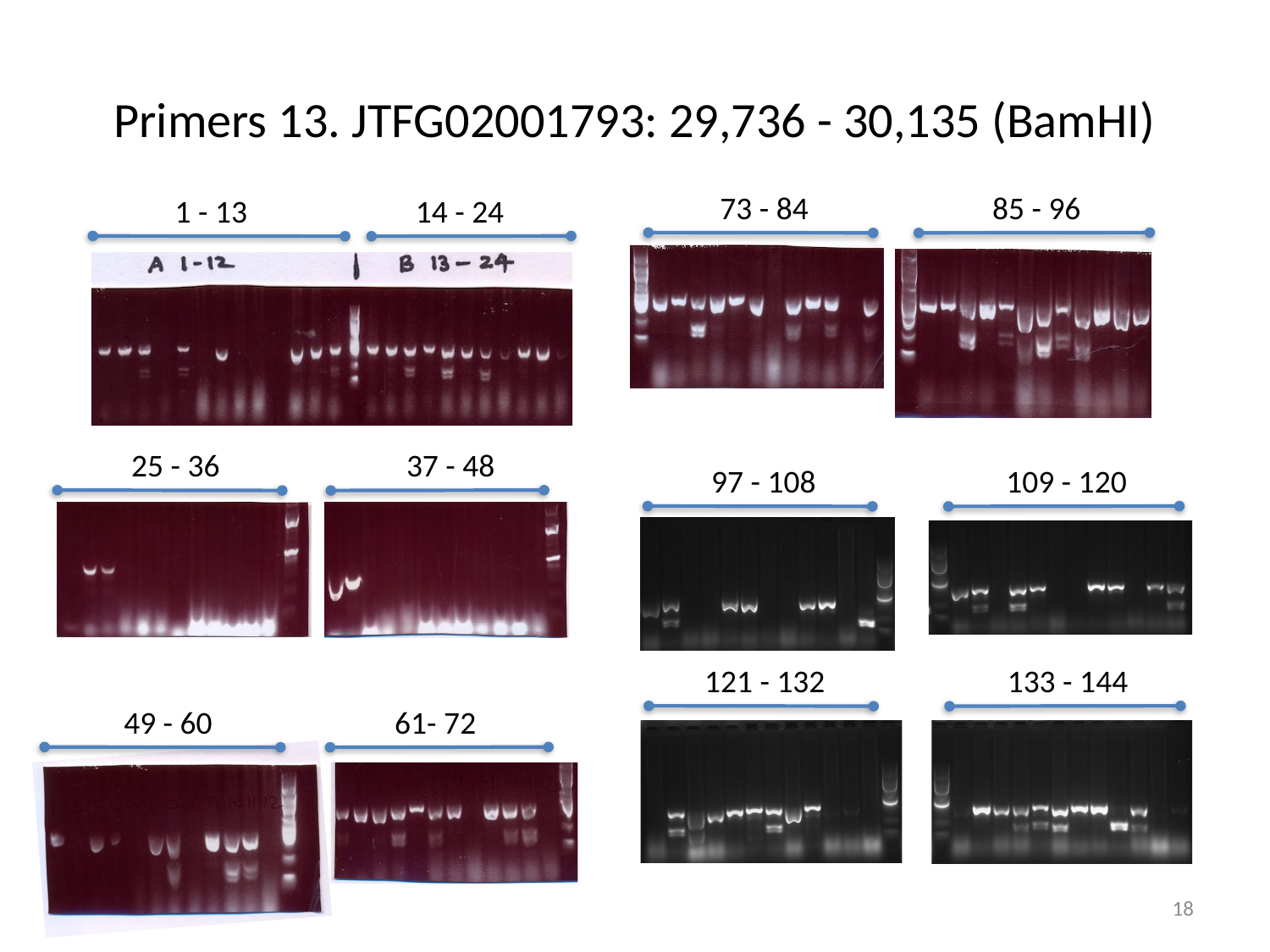

# Primers 13. JTFG02001793: 29,736 - 30,135 (BamHI)
73 - 84
85 - 96
1 - 13
14 - 24
25 - 36
37 - 48
97 - 108
109 - 120
121 - 132
133 - 144
49 - 60
61- 72
18

## Slide 19
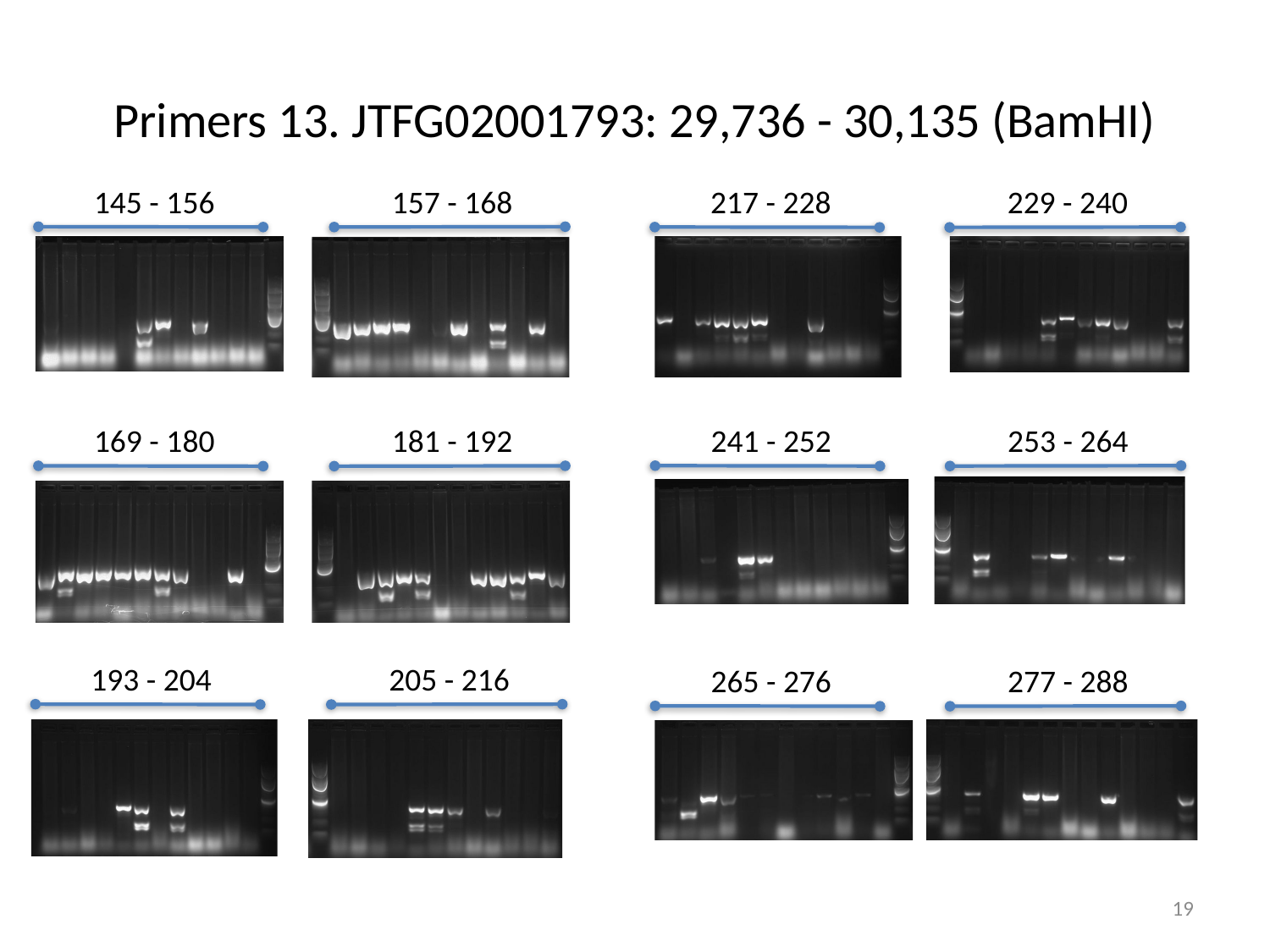

# Primers 13. JTFG02001793: 29,736 - 30,135 (BamHI)
145 - 156
157 - 168
217 - 228
229 - 240
241 - 252
169 - 180
253 - 264
181 - 192
193 - 204
205 - 216
265 - 276
277 - 288
19

## Slide 20
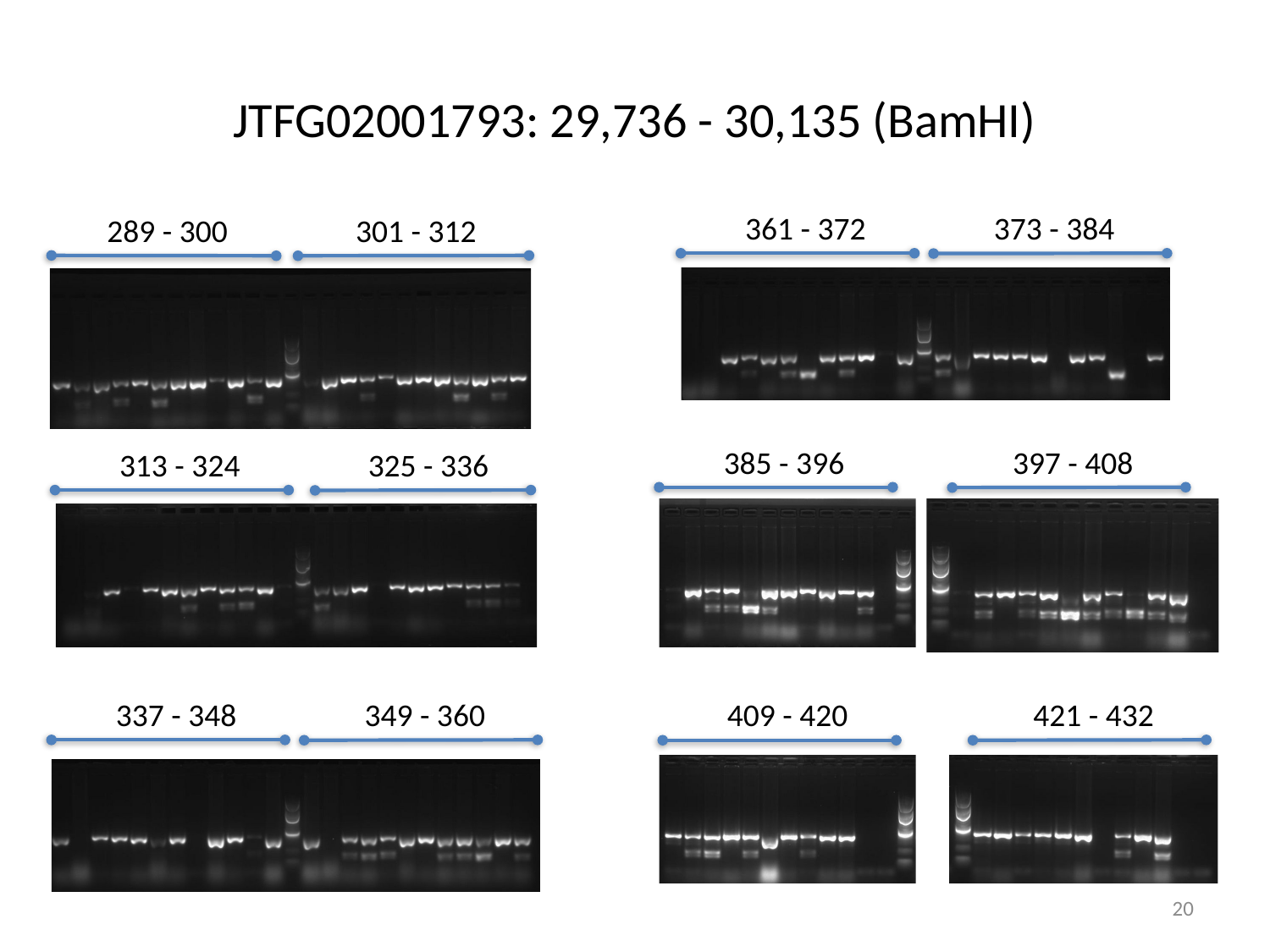

# JTFG02001793: 29,736 - 30,135 (BamHI)
361 - 372
373 - 384
289 - 300
301 - 312
385 - 396
397 - 408
313 - 324
325 - 336
337 - 348
349 - 360
409 - 420
421 - 432
20

## Slide 21
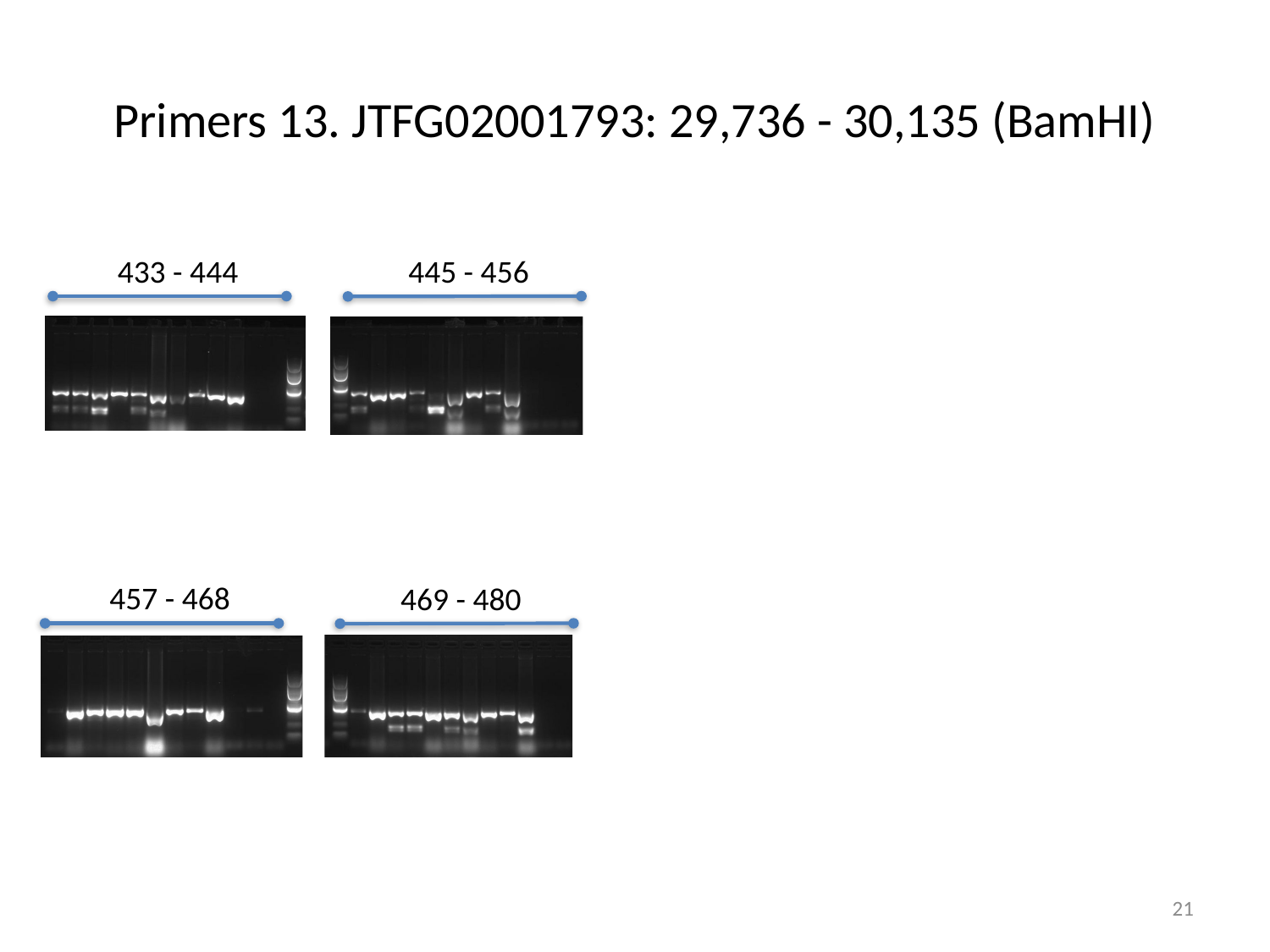

# Primers 13. JTFG02001793: 29,736 - 30,135 (BamHI)
433 - 444
445 - 456
457 - 468
469 - 480
21
