# Supplementary figures and images for: Genome sequence data from 17 accessions of Ensete ventricosum, a staple food crop for millions in Ethiopia
Source: Data Brief. 2018 Mar 11;18:285–93. doi: 10.1016/j.dib.2018.03.026 (PMC5996239; doi:10.1016/j.dib.2018.03.026)

## Slide 1
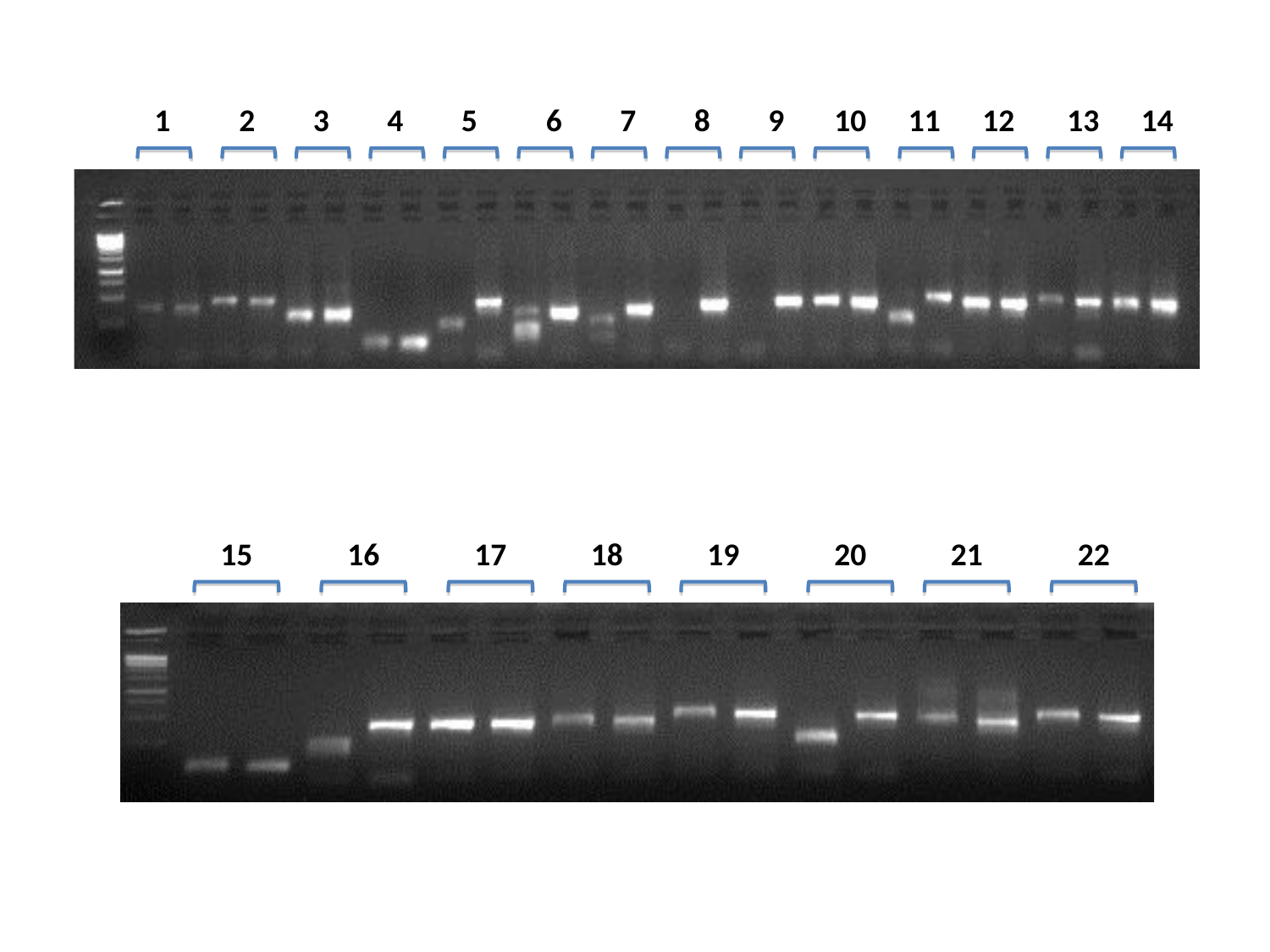

1
2
3
4
5
6
7
8
9
10
11
12
13
14
15
16
17
18
19
20
21
22

Supplement: Supplementary file 4 — Supplementary material [file mmc4.ppt]
